# Orchestrating the frontline: HDAC3-miKO recruits macrophage reinforcements for accelerated myelin debris clearance after stroke

**DOI:** 10.7150/thno.103449

**Published:** 2025-01-01

**Authors:** Jiaying Li, Chenran Wang, Yue Zhang, Yichen Huang, Ziyu Shi, Yuwen Zhang, Yana Wang, Shuning Chen, Yiwen Yuan, He Wang, Leilei Mao, Yanqin Gao

**Affiliations:** 1 State Key Laboratory of Medical Neurobiology, MOE Frontiers Center for Brain Science, and Institutes of Brain Science, Fudan University, Shanghai, China.; 2 Institute of Science and Technology for Brain-Inspired Intelligence, Fudan University, Shanghai, China.

**Keywords:** macrophages, myelin debris, chemotaxis, HDAC3-miKO, white matter repair

## Abstract

***Rational:*** White matter has emerged as a key therapeutic target in ischemic stroke due to its role in sensorimotor and cognitive outcomes. Our recent findings have preliminarily revealed a potential link between microglial HDAC3 and white matter injury following stroke. However, the mechanisms by which microglial HDAC3 mediates these effects remain unclear.

***Methods****:* We generated microglia-specific HDAC3 knockout mice (HDAC3-miKO). DTI, electrophysiological technique and transmission electron microscopy were used to assess HDAC3-miKO's effects on white matter. RNA sequencing, flow cytometry, immunofluorescence staining and *ex vivo* phagocytosis assay were conducted to investigate the mechanism by which HDAC3-miKO ameliorated white matter injury. Macrophage depletion and reconstitution experiments further confirmed the involvement of macrophage CCR2 in the enhanced white matter repair and sensorimotor function in HDAC3-miKO mice.

***Results****:* HDAC3-miKO promoted post-stroke oligodendrogenesis and long-term histological and functional integrity of white matter without affecting early-stage white matter integrity. In the acute phase, HDAC3-deficient microglia showed enhanced chemotaxis, recruiting macrophages to the infarct core probably by CCL2/CCL7, where dMBP-labelled myelin debris surged and coincided with their infiltration. Infiltrated macrophages outperformed resident microglia in myelin phagocytosis, potentially serving as true pioneers in myelin debris clearance. Although macrophage phagocytosis potential was similar between HDAC3-miKO and WT mice, increased macrophage numbers in HDAC3-miKO accelerated myelin debris clearance. Reconstitution with CCR2-KO macrophages in HDAC3-miKO mice slowed this clearance, reversing HDAC3-miKO's beneficial effects.

***Conclusions****:* Our study demonstrates that HDAC3-deficient microglia promote post-stroke remyelination by recruiting macrophages to accelerate myelin debris clearance, underscoring the essential role of infiltrated macrophages in HDAC3-miKO-induced beneficial outcomes. These findings advance our understanding of microglial HDAC3's role and suggest therapeutic potential for targeting microglial HDAC3 in ischemic stroke.

## 1. Introduction

Stroke remains a leading cause of death and disability worldwide. Unfortunately, previous therapeutic strategies directly targeting neuronal survival ended in complete failure 1, while an increasing number of studies suggested white matter as a promising target for ischemic stroke 2-6 due to its close association with sensorimotor and cognitive impairment 7-11. Indeed, while more vulnerable to ischemia compared to gray matter 12, the white matter injury process (i.e. demyelination and axonal breakup) remains reversible to some extents, unlike the irretrievable neuronal loss after ischemic stroke 13. It is also clear that the demyelinated axons are unable to efficiently perform neuronal signal transduction and function after ischemic stroke even when the neuronal somas are protected 14. Furthermore, disrupting the remyelination process after stroke also reduces neuronal survival and functional recovery after ischemic stroke 15. Of note, the clearance of myelin debris from the site of demyelination is known to be one of the prerequisites for remyelination 16. However, the pattern and destiny of myelin debris after ischemic stroke remain largely unknown. Of note, in the necrotic core of the subacute infarct, myelin debris was found to be present in CD68^+^ microglia/macrophages 17. Although less frequently discussed in the field of ischemic stroke, the important role of microglia/macrophages in the removal of myelin debris has been well recognized in various demyelination diseases, including multiple sclerosis or spinal cord injury 18-21.

As a member of class I histone deacetylases (HDACs) family, HDAC3, which composes a co-regulator complex with NCoR1 (nuclear receptor corepressor 1) and SMRT (silencing mediator of retinoic acid and thyroid hormone receptor), plays an important role in chromatin modification and thereby the transcriptional regulation of genes 22, 23. In the recent decade, HDAC3 has been reported to be implicated in neurodevelopment and various neurological diseases 24. Specifically, in the developing brain, inhibiting HDAC3 promoted the myelination process of Schwann cells and therefore facilitated peripheral myelin growth 25-27. In demyelination diseases, HDAC3 inhibitors were also found to promote the remyelination of oligodendrocytes 28, 29. Notably, according to our previous study, in the context of ischemic stroke, HDAC3 was upregulated exclusively in microglia but not in other neural cell types, suggesting microglial HDAC3 as an indispensable molecule in stroke pathology^30^. Indeed, our further investigation revealed that deletion of microglial HDAC3 decreased the SMI32/MBP ratio 35 days after stroke, which was highly correlated with the ameliorated sensorimotor behavioral outcomes 30. These results provided preliminary evidence demonstrating the association between microglial HDAC3 and de-/re-myelination process after ischemic stroke. However, the mechanism by which microglial HDAC3 regulated this essential process was still elusive.

In this study, we demonstrated that HDAC3-miKO played a reparative but not protective role in post-stroke white matter. Indeed, HDAC3-miKO promoted post-stroke oligodendrogenesis and long-term histological and functional integrity of white matter. By RNA-seq, we found that HDAC3-deficient microglia displayed enhanced chemotaxis as manifested in the increased expression of chemotaxis factors including monocyte chemoattractant CCL2 and CCL7 in the acute phase, recruiting macrophage reinforcements to the infarct core, which was coincided with the occurrence of myelin debris that was exclusively confined to the infarct core. While no difference was found in macrophages between HDAC3-miKO and WT mice in terms of their myelin phagocytosis potential, the clearance of myelin debris was accelerated in HDAC3-miKO due to the increased number of macrophages. Correspondingly, inhibiting CCR2, the receptor for CCL2/CCL7 hindered the accelerated clearance of myelin debris and reversed the beneficial effects of HDAC3-miKO. In conclusion, our study uncovered the entanglement between brain-resident microglia and the infiltrated macrophages in terms of the post-stroke white matter for the first time, advancing the understanding of the role of microglial HDAC3 and highlighting the therapeutic potential of targeting microglial HDAC3 in ischemic stroke.

## 2. Results

### 2.1. HDAC3-miKO occupies a reparative but not a protective role in post-stroke white matter

**Figure 1:**
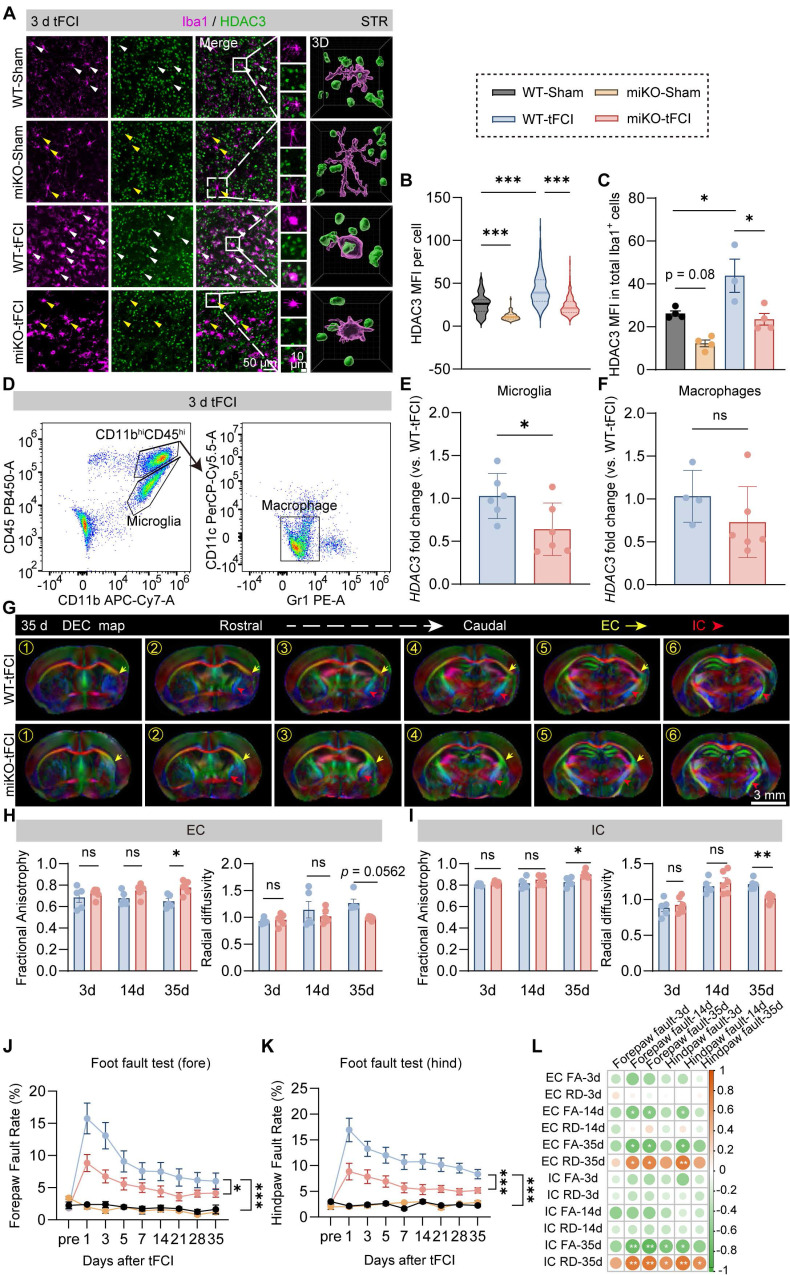
** HDAC3-miKO occupies a reparative but not a protective role in post-stroke white matter.** (**A**) Representative images of Iba1/HDAC3 immunostaining in the peri-infarct striatum (STR) 3 days after tFCI. White arrows indicate HDAC3+ microglia/macrophages. Yellow arrows indicate HDAC3- microglia/macrophages. 3D rendering was performed on the cell indicated by the white boxes to depict HDAC3 expression in Iba1+ cells. (**B**) Quantification of the mean fluorescence intensity (MFI) of HDAC3 in each Iba1+ cell. (**C**) Quantification of HDAC3 MFI in all Iba1+ cells in each animal. Each dot indicates an animal. n = 3-4 mice/group. (**D**) Gating strategy for flow-sorted microglia (CD11b^+^CD45^int^) and infiltrated macrophages (CD11b^+^CD45^+^Gr1^-^CD11c^-^). (**E&F**) RNA expression level of *Hdac3* in flow-sorted microglia (E, n = 6/group) or macrophages (F, n = 4 for WT-tFCI; n = 6 for miKO-tFCI). (**G**) DEC map presented from rostral planes to caudal planes was used to visualize *ex vivo* DTI on day 35 after tFCI. (**H&I**) FA or RD value on day 3 and day 14 in EC area (H) and IC area (I). n = 5/group. (J&K) The foot fault rate for fore paws (**J**) and hind paws (K). n = 8 for WT-Sham; n = 5 for miKO-Sham; n = 12 for WT-/miKO-tFCI. (**L**) Correlation matrix between fore/hind paw fault rates and FA/RD in EC/IC on day 3/14/35. The color and the area of circles indicate the (absolute) value of correlation coefficients. n = 5 for WT-tFCI; n = 6 for miKO-Sham. All data are presented as means±SEM. Data were analyzed using (B-C) one-way ANOVA followed by Bonferroni's post hoc, (E) unpaired two-tailed Student's t test, (F) Mann-Whitney test, and (H-K) two-way ANOVA followed by Bonferroni's post hoc, or (L) Pearson correlation. *p < 0.05, **p < 0.01, ***p < 0.001, ns: no significance, as indicated.

To define the impact of microglial HDAC3 on post-stroke white matter, we used our previously developed HDAC3 conditional knockout mouse models, which were bred from HDAC3^loxp/loxp^ and CX3CR1^CreER^ mice (Figure S1A, left panel) 30. As our previous study demonstrated 30, these transgenic mice could achieve that microglia-specific knockout of HDAC3 (HDAC3-miKO) without deleting HDAC3 in CX3CR1-expressing macrophages at least one month after tamoxifen administration (Figure S1A, right panel). After one month of tamoxifen injection, we used transient focal cerebral ischemia (tFCI) to induce a comparable decrease of cerebral blood flow in HDAC3-miKO mice and WT mice during the ischemia stage, as detected by laser Doppler flowmetry (Figure S1B). Immunofluorescence staining revealed that HDAC3, which was broadly expressed in both sham and tFCI brain, existed in the nuclear of Iba1^+^ microglia/macrophages (Figure 1A). Three days after tFCI, the expression of HDAC3 in microglia/macrophage was elevated, while HDAC3-miKO showed significantly decreased HDAC3 expression in Iba1^+^ microglia/macrophage (Figure 1B&C). To confirm the specificity of HDAC3 knockout in microglia and exclude the effects of HDAC3-miKO on HDAC3 expression in infiltrated macrophages after tFCI, we further utilized FACS to isolate macrophages (CD11b^+^CD45^hi^Gr1^-^CD11c^-^) and microglia (CD11b^+^CD45^int^) from the tFCI brains (Figure 1D) according to the previous study 31. As expected in Figure S1A, our results revealed that microglia (Figure 1E) showed significantly decreased RNA level of *Hdac3* in HDAC3-miKO while *Hdac3* expression was unchanged in infiltrated macrophages (Figure 1F). Of note, HDAC3-miKO did not show any difference in NeuN^+^ number in the peri-infarct striatum, suggesting that HDAC3-miKO did not directly protect the brain tissue at the onset of ischemic stroke (Figure S1C&D).

While our previous study has preliminarily indicated the potential role of HDAC3-miKO in white matter injury by MBP/SMI32 immunostaining 30, further investigation is still needed to determine how exactly HDAC3-miKO exerts its role in post-stroke white matter, either protective or reparative. Since diffusion tensor imaging (DTI) is able to imprint white matter tract integrity in detail and therefore provide useful insight into the de-/re-myelination process after stroke 32, we thus first used DTI to detect the white matter structural integrity *in vivo* on days 3 and 14 and *ex vivo* on day 35 (Figure 1G). For DTI, the value of fractional anisotropy (FA) reflects the fine structure of white matter. Increase of FA is coincident with white matter reorganization in the recovery region of cerebral tissue 33. The value of radial diffusivity (RD) indicates the extent of demyelination 34. Of interest, while no difference was detected on both day 3 and day 14 in two tFCI groups, a significant increase in FA values and a significant decrease in RD values were observed in both external capsule (EC) and internal capsule (IC) in the miKO-tFCI group on day 35 (Figure 1H&I), especially in some specific planes (Figure S2). This time-dependent difference indicated that HDAC3-miKO exerted reparative but not protective effects on the tFCI-induced white matter injury.

Consistent with the DTI results, HDAC3-miKO showed long-term sensorimotor improvement as revealed by foot fault test (Figure 1J&K). Of note, consistent with previous studies highlighting the close association between white matter injury and stroke outcomes 32, our behavior results were also highly correlated with the FA and RD values in both EC and IC of DTI on day 35 (Figure 1L), further supporting the pro-repair effect of HDAC3-miKO on post-stroke white matter as our previous preliminary evidence has indicated 30. We also observed the trend of improved behavioral performance as early as 1 day after tFCI. This may be due to factors beyond white matter injury, potentially involving early-stage changes in gray matter or microglial activation as reported in our previous study 30.

### 2.2. HDAC3-miKO promotes oligodendrogenesis and improves long-term histological and functional integrity of white matter after stroke

**Figure 2:**
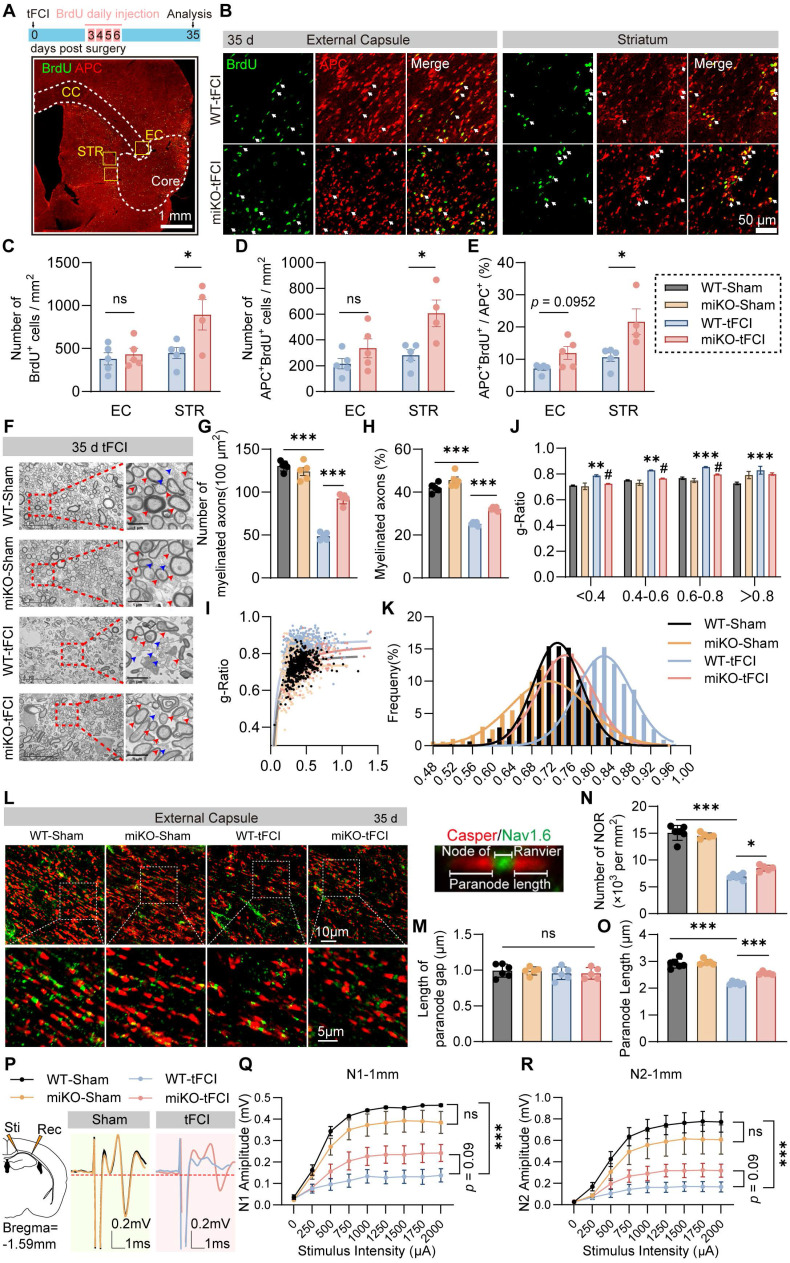
** HDAC3-miKO promotes oligodendrogenesis and improves long-term histological and functional integrity of white matter after stroke.** (**A**) Time line for BrdU injection and representative large images of BrdU/APC immunofluorescence. The yellow boxes indicated the locations where the BrdU/APC immunofluorescence images (B) were taken in the peri-infarct region of STR or EC on day 35 after tFCI. (**B**) Representative group-wise images of BrdU/APC immunofluorescence. White arrows indicated BrdU^+^APC^+^ newly-generated oligodendrocytes. (**C-E**) Quantification of the number of BrdU^+^ cells (C), APC^+^BrdU^+^ cells (D), and the percentage of APC^+^BrdU^+^ cells to the total APC^+^ cells (E). n = 5 for WT-tFCI; n = 6 for miKO-tFCI. (**F**) Representative electron micrographs. Red arrows indicated myelinated axons and blue axons indicated unmyelinated axons. (**G-K**) Quantification of the number of myelinated axons per 100 μm2 (G), the percentage of myelinated axons to the total axons (H), scatter plot of g-ratio (I), group-wise comparison of g-ratio in different scales (J), and frequency histogram of g-ratio (K). n = 5/group. (**L**) Representative images of Caspr/Nav1.6 immunofluorescence staining in the peri-infarct EC 35 days after tFCI. The right sub-image indicated an intact NOR, demonstrating the length of paranode gap and paranode length. (**M-O**) Quantification of the length of paranode gap (M), the number of NOR (N), and the paranode length (O). n = 6 for WT-Sham/-tFCI; n = 5 for miKO-Sham/-tFCI. (**P**) Schematic diagram indicating the stimulating site (Sti) and recording site (Rec) for CAPs recording in the EC at Bregma -1.59 mm and group-wise visualization of CAPs demonstrating N1 or N2 amplitudes. (**Q&R**) Group-wise comparison of N1 (Q) and N2 (R) amplitudes under different stimulus intensity. n = 5 for WT-Sham; n = 6 for miKO-Sham; n = 7 for WT-/miKO-tFCI. All data are presented as means±SEM. Data were analyzed using (C-E) unpaired two-tailed Student's t test, Mann-Whitney test, or (G&H, J, M-O) one-way ANOVA followed by Bonferroni's post hoc or (Q&R) two-way ANOVA followed by Bonferroni's post hoc. *p < 0.05, ***p < 0.001, WT -tFCI *vs.* WT-Sham or as indicated; ^#^*p* < 0.05, miKO-tFCI *vs.* WT-tFCI, ns: no significance, as indicated.

Since the repair of white matter injury is marked by successful regeneration of oligodendrocytes, we further used immunostaining of BrdU, a marker for newly generated cells, and APC, a marker for mature oligodendrocytes, to investigate whether HDAC3-miKO promotes oligodendrogenesis. We administered BrdU from 3 days to 6 days after tFCI at the peak of proliferation of oligodendrocyte precursor cells (OPCs) and conducted immunofluorescence 35 days after tFCI to label newly generated oligodendrocytes (Figure 2A) according to a previous study 35. Apparently, stroke spontaneously induced robust generation of new cells in the penumbral EC and STR, with oligodendrocytes accounting for about a half (Figure 2B). Deficiency of microglial HDAC3 promoted the overall cell proliferation in the STR as indicated by the improved BrdU^+^ cells (Figure 2C). Specifically, in agreement with our findings by DTI, miKO-tFCI did show increased APC^+^BrdU^+^ cells in the STR (Figure 2D) and proportion of APC^+^BrdU^+^ cells to total APC^+^ cells in both the EC and the STR (Figure 2E), further suggesting improved remyelination in miKO-tFCI mice.

Indeed, as demonstrated by TEM, we detected the ultrastructure changes of myelinated axons 35 days after tFCI (Figure 2F) and revealed that miKO-tFCI significantly reversed the decreased number or proportion of myelinated axons (Figure 2G&H). G-ratio directly reflects the thickness of axon-enwrapping myelin sheath with lower g-ratio indicating thicker myelin sheath, serving as a highly reliable index for assessing axonal myelination 36. Our results revealed that tFCI induced higher g-ratio compared to Sham, while HDAC3-miKO remarkably diminished g-ratio, suggesting the restoration of structural integrity of white matter (Figure 2I-K).

Lengthening of the node of Ranvier (NOR) due to myelin retraction or even the loss of NOR are well known to contribute to altering the function of myelinated axons in stroke pathology 37. Thus, we employed Caspr/Nav1.6 immunostaining to detect the change of NOR (Figure 2L). Consistent with previous studies 37, the pathological condition or HDAC3-miKO still kept the paranode structure intact, as indicated by the unchanged length of paranode gap (Figure 2M). However, tFCI reduced both number of NOR or the paranode length, while miKO-tFCI increased number of NOR and paranode length to some extents (Figure 2N&O), suggesting the improved function of myelinated axons in miKO-tFCI. Indeed, the performance in the foot fault test was correlated with both the NOR number (Figure S3A&B) and the paranode length (Figure S3C&D).

To verify whether the aforementioned structural change contributes to functional integrity of white matter, we performed electrophysiological recordings by detecting composite action potentials (CAPs) in the CC 35 days after ischemic stroke. CAPs recordings reflect the combined conduction of both unmyelinated and myelinated fibers in the CC. The amplitudes of the faster action potential N1 and slower action potential N2 represent the conduction potential of the myelinated and unmyelinated fibers, representatively (Figure 2P). We demonstrated that HDAC3-miKO prevented the stroke-induced decrease in N1 as well as N2 amplitude almost significantly (Figure 2Q&R). Correlation analysis revealed that N1 amplitude was associated with the forepaw fault rate on day 35 (Figure S3E), further suggesting that the axonal conductive speed was closely related to behavioral performance.

Collectively, HDAC3-miKO promoted oligodendrogenesis of the post-stroke brain and restored structural and functional integrity of white matter long-term after tFCI.

### 2.3. RNA-seq reveals increased chemotaxis in HDAC3-deficient microglia

Of note, due to their early-stage heterogeneous nature, microglia act as a double-edge sword for the later remyelination process after stroke. On one hand, microglia secrete inflammatory factors that hinder oligodendrocyte function 38. On the other hand, pro-regenerative microglia could also promote remyelination by removing myelin debris and releasing regenerative factors 39. Therefore, it is reasonable that the early-stage HDAC3-deficient microglia may have exerted determinant effects on the stroke-induced white matter injury. In this case, we isolated CD45^int^CD11b^+^ microglia on day 3 after tFCI and performed bulk RNA-seq to investigate the mechanism by which HDAC3-miKO modulated post-stroke white matter.

**Figure 3:**
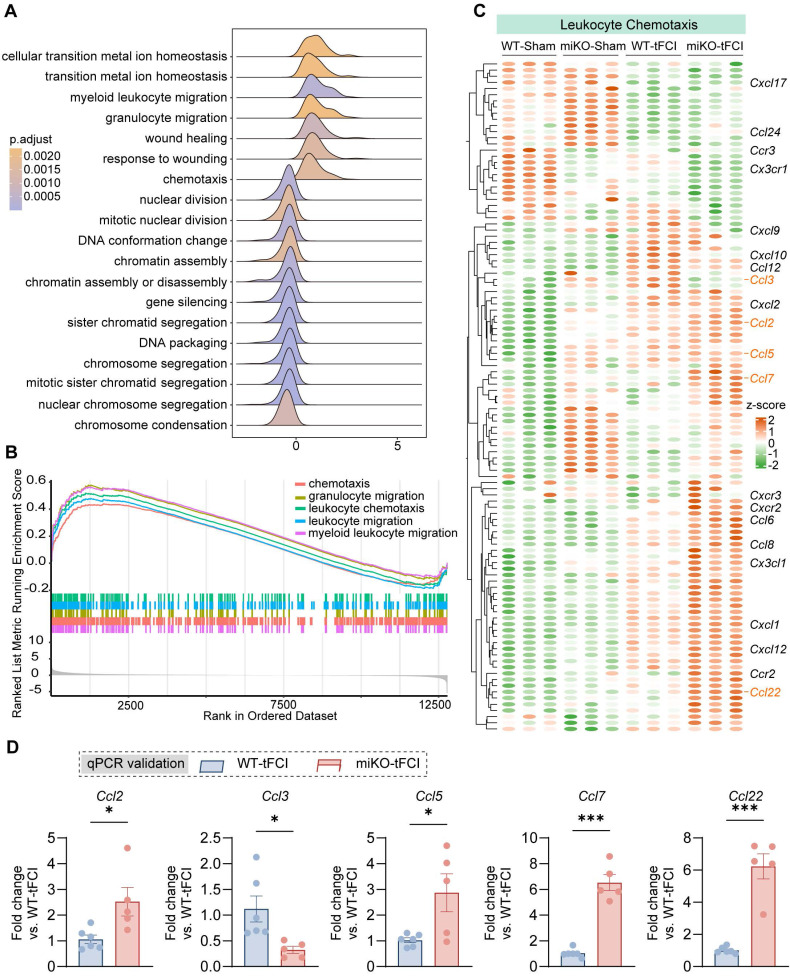
** RNA-seq reveals increased chemotaxis in HDAC3-deficient microglia.** (**A**) Ridge plot visualizing GSEA results (miKO-tFCI vs. WT-tFCI). (**B**) GSEA results showed significantly upregulated GO pathways of “chemotaxis”, “granulocyte migration”, “leukocyte migration” and “myeloid leukocyte migration” (miKO-tFCI vs. WT-tFCI). (**C**) Heatmap showed expression level of genes associated with the term “Leukocyte Chemotaxis” in microglia. Each column represents a biological replicate. (**D**) qPCR validation of representative C-C chemokine genes whose expression significantly changed in miKO-tFCI vs. WT-tFCI. n = 6 for WT-tFCI; n = 5 for miKO-tFCI. All data are presented as means±SEM. Data were analyzed using unpaired two-tailed Student's t test. *p < 0.05, ***p < 0.001, as indicated.

In our previous study, we have identified massive DEGs between groups (4345 DEGs in WT-tFCI vs. WT-Sham, 1310 DEGs in miKO-tFCI vs. WT-tFCI, and 2288 DEGs in miKO-Sham vs. WT-Sham), suggesting a considerable difference induced by either tFCI or HDAC3-miKO 30. Then we used the DEGs generated from miKO-tFCI *vs.* WT-tFCI for the downstream gene set enrichment analysis (GSEA) on the basis of GO database. Surprisingly, in addition to the downregulated proliferation-associated terms including “sister chromatid segregation”, “mitotic sister chromatid segregation” and “nuclear chromosome segregation” that have been carefully explored in our previous study 30, the GSEA results also uncovered the simultaneously upregulated chemotaxis-associated terms, including “chemotaxis”, “myeloid leukocyte migration”, “granulocyte migration”, “leukocyte chemotaxis”, and “leukocyte migration” (Figure 3A&B). Correspondingly, depleting microglial HDAC3 further elevated the expression of numerous stroke-induced upregulated genes associated with leukocyte chemotaxis (Figure 3C). Among these genes, we noticed that C-C chemokines including *Ccl2, Ccl3, Ccl5, Ccl7* and *Ccl22* showed remarkable upregulation in HDAC3-deficient microglia, as validated by qPCR (Figure 3D). These chemokines are engaged in recruitment of multiple types of immune cells, suggesting the possibility that HDAC3-deficient microglia directly called for reinforcements of peripheral immune cells while their own expansion was disrupted.

### 2.4. HDAC3-deficient microglia recruit macrophage reinforcements to the infarct core

**Figure 4:**
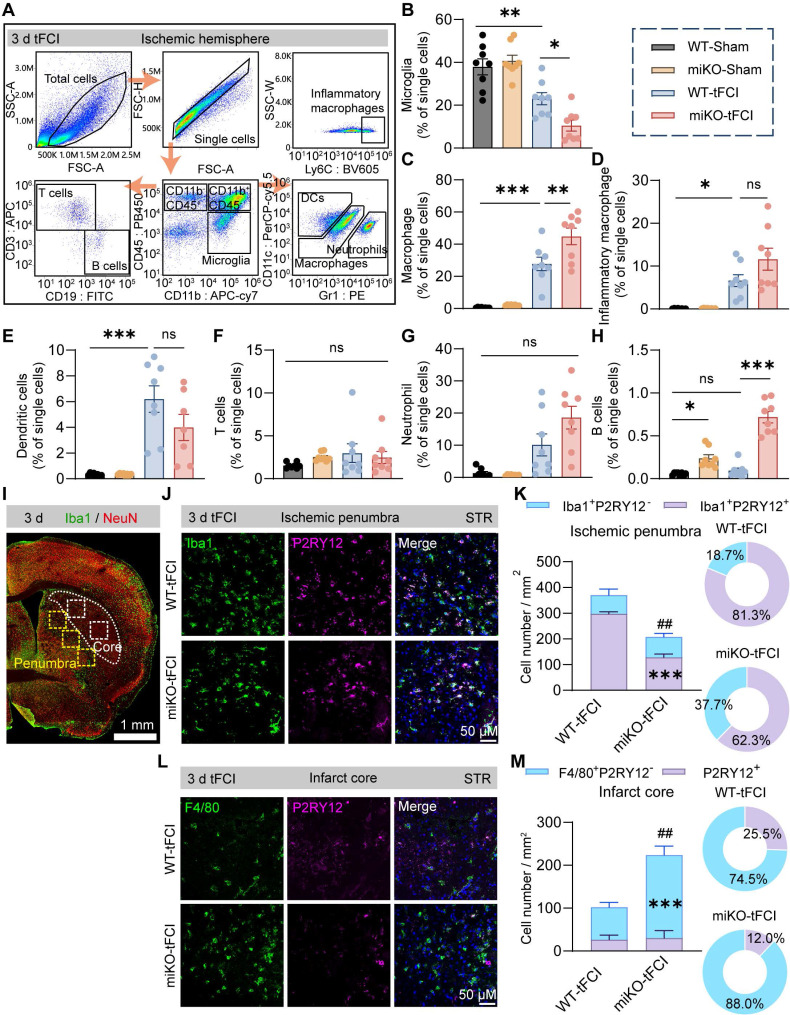
** HDAC3-deficient microglia recruit macrophage reinforcements to the infarct core.** (**A**) Macrophages (CD11b^+^CD45^hi^Ly6C^+^), neutrophils (CD11b^+^CD45^hi^CD11c^-^Gr1^+^), dendritic cells (DCs) (CD11b^+^CD45hiCD11c^+^Gr1^-^), T cells (CD11b^-^CD45^hi^CD3^+^), B cells (CD11b^-^CD45^hi^CD19^+^) and microglia (CD11b^+^CD45^int^). (**B-H**) The percentage of these cells to single cells, respectively. n = 8/group. (**I**) Representative large image of Iba1/NeuN immunostaining, which indicated the infarct core and penumbra region photographed for Iba1/F4/80/P2RY12 staining (J-M). (**J&K**) Representative images of Iba1/P2RY12 immunostaining (J) and quantification of the number of Iba1^+^P2RY12^-^ cells and Iba1^+^P2RY12^+^ cells (K) in the ischemic penumbra 3 d after tFCI. The right panel showed the proportion of Iba1^+^P2RY12^-^ cells or Iba1^+^P2RY12^+^ cells to the overall Iba1^+^ cells. (**L&M**) Representative images of F4/80/P2RY12 immunostaining (L) and quantification of the number of F4/80^+^P2RY12^-^ cells and P2RY12^+^ cells (M) in the ischemic core 3 d after tFCI. The right panel showed the proportion of F4/80^+^P2RY12^-^ cells or P2RY12^+^ cells to the overall F480^+^ or P2RY12^+^ cells. n = 4 for WT-tFCI; n = 5 for miKO-tFCI. All data are presented as means±SEM. Data were analyzed using (B-E) one-way ANOVA followed by Bonferroni's post hoc, (F-H) Kruskal-Wallis test followed by Dunn's post hoc test or (K&M) unpaired two-tailed Student's t test. *p < 0.05, **p < 0.01, ***p < 0.001, ns: no significance, as indicated (B-H). ***p < 0.001 for comparison of Iba1^+^P2RY12^+^ cell number between miKO-tFCI and WT-tFCI. ^##^*p* < 0.01 for comparison of the overall Iba1+ cells (K). ***p < 0.001 for comparison of F4/80^+^P2RY12^-^ cell number between miKO-tFCI and WT-tFCI. ^##^*p* < 0.01 for comparison of the overall F480^+^ or P2RY12^+^ cells (M).

Therefore, to determine the certain type of infiltrated immune cells that were augmented in HDAC3-miKO mice, we proceed to perform flow cytometry to analyze the infiltrated cells 3 days after tFCI using the gating strategy of T cells (CD11b^-^CD45^hi^CD3^+^), B cells (CD11b^-^CD45^hi^CD19^+^), dendritic cells (CD11b^+^CD45^hi^CD11c^+^Gr1^-^), neutrophils (CD11b^+^CD45^hi^CD11c^-^Gr1^+^), microglia (CD11b^+^CD45^int^), macrophages (CD11b^+^CD45^hi^) and inflammatory macrophages (CD11b^+^ CD45^hi^ Ly6C^+^) (Figure 4A). Consistent with our previous study^30^, HDAC3 deficiency further decreased microglial number after tFCI by over a half (Figure 4B). In addition, tFCI remarkably induced the recruitment of macrophages (including inflammatory macrophages) and DCs but not neutrophils, T cells or B cells, in agreement with existing reports (Figure 4C-H) 40. In our study, neither tFCI nor HDAC3-miKO induced remarkable immune response in the peripheral blood or spleen (Figure S4). Notably, among all these immune cells, HDAC3-miKO recruited significantly more macrophages compared to WT, while the numbers of other infiltrated cells remained unchanged (Figure 4C). However, the number of inflammatory macrophages did not show significant change as the overall macrophages did (Figure 4D). Another interesting fact showed that HDAC3-miKO also facilitated recruitment of B cells even into the Sham-induced brains and also into the tFCI-induced brain to a greater extent 3 days after tFCI (Figure 4H) when infiltration of B cells was not observed. More investigations involving more time points regarding this phenomenon were needed in the future study. Considering that B cells did not infiltrate into the brain and remained a small population even in HDAC3-miKO group at this early stage, in this study, we still aimed at macrophages, which have been widely reported to exert an important but controversial role in stroke pathology 41.

It is well known that infiltrated macrophages share very similar signatures and functions with brain-resident microglia under pathological conditions 42. However, in recent years, owing to the emerging techniques that can well distinguish macrophages from microglia, growing studies have highlighted the different roles between macrophages and microglia. Given that the pathological environment differed according to the distance to the infarct core, understanding the distribution of macrophage reinforcements may help us further investigate their role. In this case, we employed immunofluorescence staining of P2RY12, a marker for *bona fide* microglia, and F4/80, a marker for activated microglia and infiltrated macrophages, to determine the macrophages distribution in the contralateral hemisphere, peri-infarct region and infarct core. As expected, Iba1^+^P2RY12^-^ infiltrated macrophages were not observed in the contralateral hemisphere (Figure S5). In the peri-infarct STR, Iba1^+^P2RY12^+^ brain-resident microglia predominated the region in terms of the overall Iba1^+^ cells in both two groups, and miKO-tFCI mice had significantly fewer Iba1^+^ microglia/macrophages, mainly attributed to a significant reduction in the number of microglia (Figure 4I-K), supporting our previous study that HDAC3-miKO inhibited microglial proliferation 30. Of note, the absolute number of infiltrated macrophages in the peri-infarct region did not show any significant change (Figure 4K). Unlike the peri-infarct region, in the infarct core, macrophages far outnumbered microglia, in agreement with previous studies indicating robust loss of microglia and the repopulation by infiltrated macrophages in the infarct core (Figure 4L-M) 43. Interestingly, in this region, HDAC3-miKO induced significant increase in the number of infiltrated macrophages while the number of microglia remained unchanged (Figure 4M). Taken together, these findings implied that HDAC3-miKO resulted in more macrophages infiltration into the infarct core but not into the penumbra area.

### 2.5. Spatiotemporal pattern of myelin debris distribution after stroke

**Figure 5:**
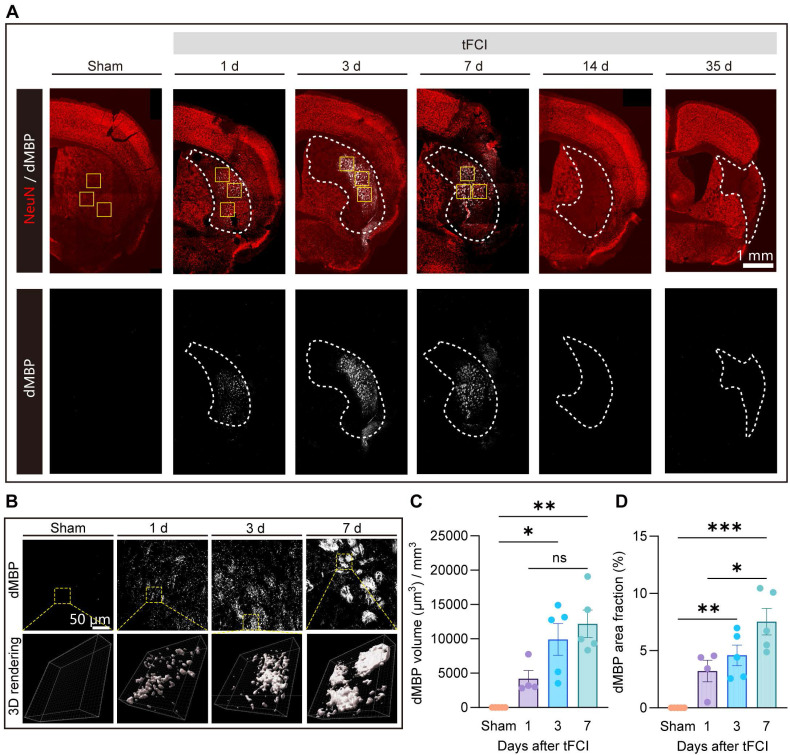
** Spatiotemporal pattern of myelin debris distribution after stroke.** (**A**) Representative large images of dMBP/NeuN immunofluorescence staining on Sham group or on day 1,3,7,14,35 after tFCI. The white dashed lines outlined the infarct core with neuronal loss. The following images (**B**) were taken from where the yellow boxes indicated. (B) Representative images demonstrating dMBP signals and the 3D rendering of these signals in the infarct core. (**C**) Quantification of dMBP volume (μm3) per mm3. (**D**) Quantification of dMBP area fraction (%). n = 4 for WT-/miKO-Sham; n = 5 for WT-/miKO-tFCI. All data are presented as means±SEM. Data were analyzed using (C) one-way ANOVA followed by Bonferroni's post hoc and (D) Kruskal-Wallis test followed by Dunn's post hoc test *p < 0.05, **p < 0.01, ***p < 0.001, ns: no significance, as indicated.

The removal of myelin debris is an essential step in the remyelination process. Mononuclear phagocytic cells, including monocyte-derived macrophages and microglia, are actively implicated in the myelin debris clearance 21, 44-46. While the clearance of myelin debris has been well discussed in demyelinating diseases 21, 46, this process and the associated mechanism specific to stroke pathology are poorly understood. Recognizing the spatiotemporal pattern of myelin debris distribution after stroke is a necessary prerequisite to understanding of myelin debris clearance. Therefore, we performed immunofluorescence staining of NeuN and dMBP (a marker for degenerated myelin) on Sham and on day 1, 3, 7, 14 and 35 after tFCI (Figure 5A). We demonstrated that in the context of stroke, myelin debris was only present in the infarct core where NeuN^+^ neurons vanished on day 1, 3 and 7 (Figure 5B). Quantification of the volume or the area fraction of dMBP^+^ myelin debris exhibited that as the stroke pathology proceeded, dMBP signal augmented and remained high on both day 3 and day 7 (Figure 5C&D).

### 2.6. HDAC3-miKO accelerates myelin debris clearance by boosting macrophage recruitment without altering phagocytosis capability

Based on the above understanding that infiltrated macrophages predominantly occurred in the infarct core where myelin debris surged and that HDAC3-deficient microglia have summoned additional macrophage reinforcements, we hypothesized that HDAC3-miKO could accelerate myelin debris clearance by boosting macrophage recruitment, in this way promoting oligodendrogenesis and improving long-term histological and functional integrity of white matter after stroke.

**Figure 6:**
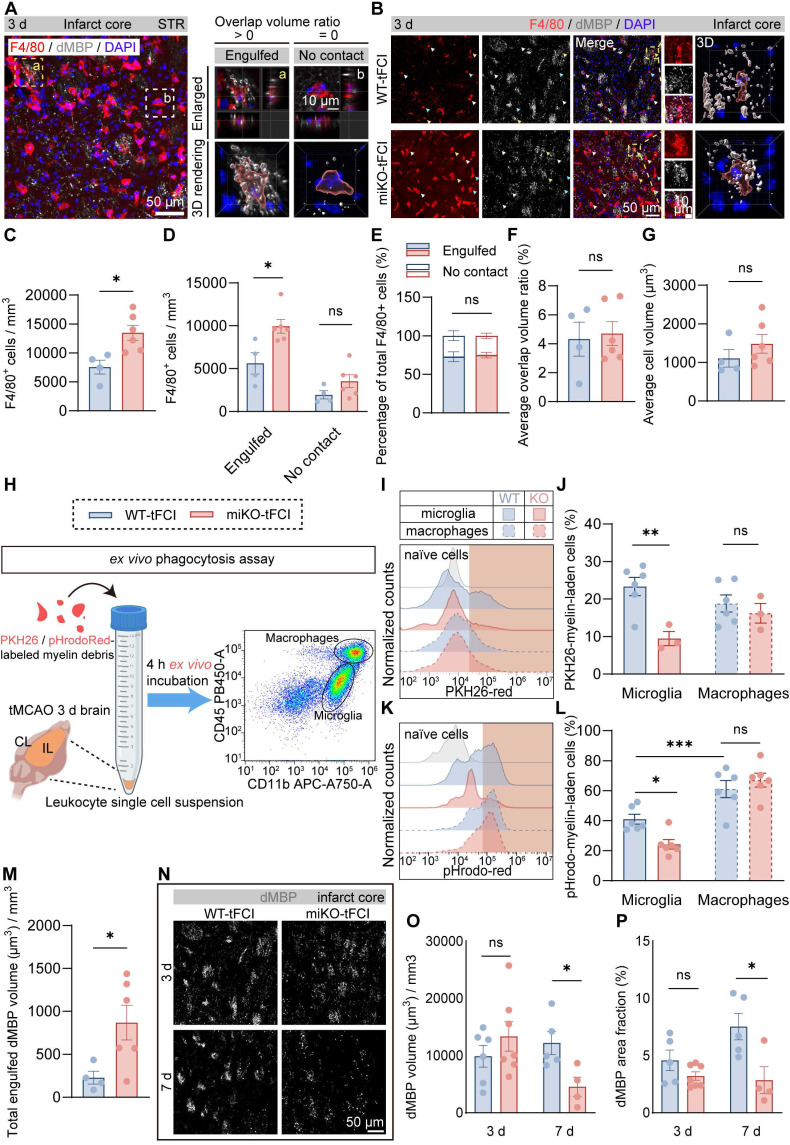
** HDAC3-miKO accelerates myelin debris clearance by boosting macrophage recruitment without altering phagocytosis capability.** (**A**) Representative image of F4/80/dMBP immunofluorescence staining, indicating the (a) “Engulfed” state (overlap volume ratio > 0) or (b) “No contact” state (overlap volume ratio = 0) of F4/80^+^ cells. (**B**) Group-wise representative images of F4/80/dMBP immunofluorescence staining and the associated 3D rendering illustrating the internalization of dMBP^+^ myelin debris by an F4/80^+^ cell. (**C**) Quantification of F4/80^+^ cell number per mm^3^. (**D&E**) Quantification of the number of F4/80^+^ “Engulfed” or “No contact” cells (D), their respective percentage to the total F4/80^+^ cells (E). (**F&G**) The average overlap volume ratio of all F4/80^+^ cells (F) and the average cell volume of all F4/80^+^ cells (G) in all the FOVs photographed. Each dot represented a mouse, 10-15 F4/80^+^ cells were quantified for each mouse. n = 4 for WT-tFCI; n = 6 for miKO-tFCI (C-G). (**H**) Schematic diagram showing the method of *ex vivo* phagocytosis assay. (**I**) Representative histograms showing PKH26-red fluorescence intensity in CD11b^+^CD45^int^ microglia or CD11b^+^CD45^hi^ macrophages, assessed by flow cytometry. (**J**) Percentage of microglia/macrophages containing PKH26 signal. n = 6 for WT-tFCI; n = 3 for miKO-tFCI. (**K**) Representative histograms showing pHrodo-red fluorescence intensity in CD11b^+^CD45^int^ microglia or CD11b^+^CD45^hi^ macrophages, assessed by flow cytometry. (**L**) Percentage of microglia/macrophages containing pHrodo-red signal. n = 6/group. (**M**) Quantification of total engulfed dMBP volume by F4/80^+^ cells in all photographed FOVs. Each dot represented a mouse. n = 4 for WT-tFCI; n = 6 for miKO-tFCI. (**N**) Representative images of dMBP immunofluorescence staining on day 3/7 after tFCI. (**O**) Quantification of dMBP volume (μm^3^) per mm^3^. (**P**) Quantification of dMBP area fraction (%).3 d: n = 6 for WT-tFCI; n = 7 for miKO-tFCI. 7 d: n = 5 for WT-tFCI; n = 4 for miKO-tFCI (O&P). All data are presented as means±SEM. Data were analyzed using (C-G, J&L and N-P) unpaired two-tailed Student's t test, Mann-Whitney test or (J&L) one-way ANOVA followed by Bonferroni's post hoc. *p < 0.05, **p < 0.01, ***p < 0.001, ns: no significance, as indicated.

Therefore, we employed 3D reconstruction of F4/80/dMBP immunostaining to quantify the phagocytosis capacity of myelin debris by F4/80^+^ cells (mostly infiltrated macrophages), as demonstrated as overlap volume ratio. We defined cells with overlap volume ratio > 0 as “Engulfed” macrophages, or otherwise “No contact” macrophages (Figure 6A). Consistent with our previous results (Figure 4M), HDAC3-miKO experienced an increase in the number of macrophages in the infarct core (Figure 6B&C). Among these cells, “Engulfed” cells accounted for the majority and increased significantly in HDAC3-miKO while “No contact” cells were comparable in two tFCI groups (Figure 6D). However, the percentage of both cell types to the total F4/80^+^ cells did not significantly change (Figure 6E), suggesting that HDAC3-miKO did not alter the phagocytosis capacity of macrophages actually. Indeed, no significant difference was observed in the average overlap volume ratio for each cell in two groups (Figure 6F). Moreover, the average cell volume, which has been reported to imply the cellular phagocytosis state, did not significantly change as well in two groups (Figure 6G).

To precisely determine the phagocytosis potential of infiltrated macrophages in two groups, we conducted an* ex vivo* phagocytosis assay as previous studies described using PKH26 or pHrodo-Red-labelled myelin debris and leukocytes collected from brains 3 days after tFCI (Figure 6H). In details, PKH26 is a lipophilic dye that was able to simply detect the uptake of myelin debris, and pHrodo-Red is a pH-sensing dye that was able to further detect the degradation of myelin debris owing to its dramatic increase in fluorescence in acidic lysosomes. After 4 h *ex vivo* incubation with myelin debris, CD11b^+^CD45^hi^ cells (mostly macrophages) in two tFCI groups displayed comparable capacity for both uptake (Figure 6I, J right panels) and degradation (Figure 6K, L right panels), in agreement with the fact that macrophages in HDAC3-miKO remained HDAC3-sufficient (Figure 1F). However, despite the unchanged phagocytosis capacity, macrophages in HDAC3-miKO still internalized more dMBP^+^ myelin debris as a whole 3 days after tFCI (Figure 6M). Given that HDAC3-miKO promoted macrophages infiltration into the infarct core, we inferred that the only reason why myelin debris was more engulfed in macrophage population in HDAC3-miKO was because of the increased number of but not the increased phagocytosis capacity of macrophages. Surprisingly, unlike macrophages, HDAC3-deficient microglia (CD11b^+^CD45^int^) showed significantly reduced capacity for both uptake and degradation of myelin debris (Figure 6J&L, left panels). Corresponding to this result, in the penumbra area, HDAC3-deficient microglia showed remarkably smaller proportion contacting with non-degraded intact myelin bundles (Figure S6). A previous study has suggested that contact with intact myelin sheath exacerbated the ischemia outcomes 47. Therefore, our result indicated that HDAC3-deficient microglia may also directly contribute to the reduced myelin damage after stroke.

To verify whether HDAC3-miKO subsequently affected the clearance of myelin debris, we further evaluated dMBP signal on day 3 and 7 after tFCI (Figure 6N), when dMBP signal remained apparent (Figure 5). Our results demonstrated that although dMBP signal did not significantly change on day 3 in two groups, HDAC3-miKO did induce more efficient clearance of myelin debris later on day 7 after tFCI (Figure 6O&P).

Taken together, our results indicated that HDAC3-miKO promoted myelin debris clearance exclusively by recruiting more macrophages without affecting their phagocytosis capacity.

### 2.7. CCR2-KO BMDMs reconstitution reverses the effects of HDAC3-miKO

**Figure 7:**
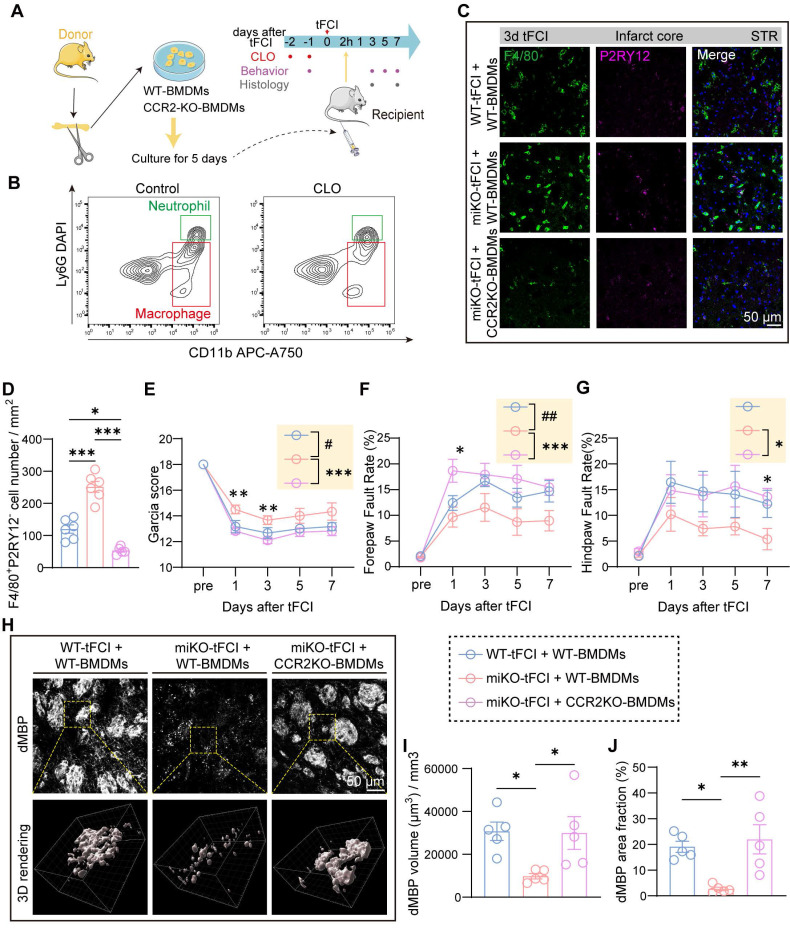
** CCR2-KO BMDMs reconstitution reduces infiltration into the brain and reverses the effects of HDAC3-miKO. (A)** Experimental design indicating the strategy for CCR2 deficency macrophages administration. **(B)** Flow cytometry showing depletion of macrophages 24 h after CLO administration. **(C-D)** Representative images of F4/80/P2RY12 immunostaining and (D) quantification of the number of F4/80^+^P2RY12^‑^ cells.** (E-G)** Sensorimotor function assessment by Garcia score (E) and foot fault test (F&G). n = 6 for WT-tFCI+WT-BMDM or miKO-tFCI+WT-BMDM; n = 11 for miKO-tFCI+CCR2 KO-BMDM. **(H)** Representative images of dMBP immunofluorescence staining and the corresponding 3D rendering in three groups. **(I)** Quantification of dMBP volume (μm^3^) per mm^3^. **(J)** Quantification of dMBP area fraction (%). All data are presented as means±SEM. Data were analyzed using (D&I-J) one-way ANOVA followed by Bonferroni's post hoc test or (E-G) two-way ANOVA followed by Bonferroni's post hoc test. Comparisons in the yellow blocks referred to the main effects between groups for the ANOVA. *p < 0.05, **p < 0.01, ***p < 0.001 for comparison between miKO-tFCI+WT-BMDM and miKO-tFCI + CCR2-KO BMDM; ^#^*p* < 0.05; ^##^*p* < 0.01 for comparison between WT-tFCI + WT-BMDM and miKO-tFCI + WT-BMDM.

To investigate the role of macrophage CCR2 in mediating HDAC3-miKO effects after tFCI, we employed a macrophage reconstitution approach (Figure 7A) based on a previous study 48. In brief, after isolation from bone marrow of WT or CCR2-KO mice (Figure S7A), bone marrow-derived macrophages (BMDMs) were cultured for 5 days for differentiation. Resident macrophages in WT and HDAC3-miKO mice were depleted by intravenous injection of clodronate (CLO) administered for two consecutive days starting 2 days prior to tFCI. Flow cytometry showed reduction of macrophages in blood after CLO injection (Figure 7B). Subsequently, BMDMs were intravenously injected 2 hours after tFCI.

Consistent with our previous reasoning, reconstitution with CCR2-KO BMDMs (miKO-tFCI + CCR2-KO BMDMs) significantly decreased macrophage recruitment to the infarct core in HDAC3-miKO mice compared to those reconstituted with WT BMDMs (miKO-tFCI + WT BMDMs) (Figure 7C&D). Despite similar weight and survival rates across the three tFCI groups (Figure S7B&C), CCR2-KO BMDMs reconstitution reversed the improved outcomes in HDAC3-miKO mice, as revealed by lower Garcia scores and poorer foot-fault test performance (Figure 7E-G).

Given that our prior results implicated recruited macrophages in facilitating myelin debris clearance within the infarct core, we evaluated the dMBP signal on day 7 post-tFCI across the three groups (Figure 7H). As expected, miKO-tFCI + WT BMDMs exhibited significantly reduced dMBP volume and area fraction compared to WT-tFCI + WT BMDMs (Figure 7I&J, blue bars vs. red bars). Notably, this reduction was reversed in miKO-tFCI mice reconstituted with CCR2-KO BMDMs (Figure 7I&J, red bars vs. pink bars).

Similarly, administration of the selective CCR2 antagonist RS504393 yielded comparable results (Figure S8). Collectively, these findings demonstrate that macrophage CCR2 mediates HDAC3-miKO-induced accelerated clearance of myelin debris, contributing to the observed beneficial outcomes.

## 3. Discussion

Over the past years, a growing body of studies has suggested that HDAC3 may serve as a therapeutic target for a variety of neurological diseases probably by modulating neuroinflammation 49-53. Consistently, our recent study has revealed that ischemic stroke induced elevation of HDAC3 in microglia specifically but not in other cell types 30. The study also preliminarily uncovered that HDAC3-miKO decreased the MBP/SMI32 ratio 35 days after tFCI 30, leading us to further question how HDAC3 was implicated in the de-/re-myelination process after stroke.

Herein, by RNA-seq, we found that in addition to diminished proliferation as reported in our previous study, HDAC3-deficient microglia also showed enhanced chemotaxis, specifically manifested by increased macrophage infiltration as revealed by flow cytometry. Considering the similar nature between macrophages and microglia, this interesting finding that infiltrated macrophages grew as brain-resident microglia declined or failed to expand is irresistibly reminiscent of a recently constructed hypothesis that circulating monocytes compete for a limited number of niches against microglia 54. When the niche is available, circulating monocytes enter the brain, differentiate into macrophages and even developed microglia-like characteristics, maintaining the brain macrophage pool and presumably performing the functions that microglia should have been responsible for 55. Indeed, our further immunofluorescence analysis revealed that F4/80^+^P2RY12^-^ infiltrated macrophages took up the infarct core where microglia were infertile, consistent with previous studies 56. Interestingly, regardless of the presence or absence of microglial HDAC3, microglia and infiltrated macrophages always firmly occupied their respective compartments, as supported by the fact that HDAC3-miKO induced increased number of macrophages only in the infarct core but not in the peri-infarct area. It appeared that HDAC3-deficient microglia orchestrated the cell composition of the post-stroke brain by recruiting more macrophage reinforcements to the infarct core. Of note, this promoted macrophages' colonization of microglia niche may be independent of the change of blood brain barrier (BBB) breakdown. Under physiological conditions, microglial depletion alone was sufficient to induce the refill by macrophages without BBB disruption 55. In the context of stroke, while BBB disruption has served as a prerequisite for macrophages infiltration, we believed that HDAC3-miKO did not aggravate BBB breakdown after stroke, given the unchanged number of other infiltrated immune cells. Then how did the macrophages sense the available niches? While the mechanism by which macrophage refill the niche with microglial loss was largely unknown 54, our RNA-seq results provided a potential candidate mechanism that HDAC3-deficient microglia themselves could secret significantly more monocyte chemoattractants CCL2 and CCL7, which may directly encourage the stationing of more macrophage reinforcements. However, it remains elusive how exactly HDAC3 deficiency regulated microglial chemokine expression. On one hand, HDAC3 plays a well-established role in modifying histone acetylation to regulate gene transcription. For example, HDAC3 has been shown to suppress H3K27ac at the promoters of chemokines such as CXCL9, CXCL10, and CXCL11, thereby directly inhibiting their expression 57. This suggests that HDAC3 could modulate microglial chemokine expression through changes in histone modifications, which directly affect chromatin structure and accessibility. On the other hand, HDAC3 also regulates non-histone acetylation, including the deacetylation of transcription factors like NF-κB and STAT3, which are crucial for the regulation of inflammation and immune responses. For instance, HDAC3 has been shown to deacetylate the NF-κB p65 subunit, suppressing its transcriptional activity and consequently affecting its role in leukocyte adhesion and chemotaxis. Similarly, HDAC3 deacetylates STAT3, a transcription factor that regulates neutrophil mobilization and MIP-2-dependent neutrophil chemotaxis. In addition, Safronova *et al.* reported that HDAC3 is involved in hypoxic repression of CCL2 by enhancing the formation of an inactive P-TEFb complex, a transcription elongation factor, which regulates RNA polymerase II elongation competence 58. General HDAC inhibitors elevated CCL2 expression in melanoma, consistent with our observation in HDAC3-deficient microglia 59. However, opposite to our results, it has been reported that HDAC inhibition decreased CCL7 expression due to the repression of PU.1, which predominantly mediates CCL7 expression 60. Therefore, although our previous study has revealed that HDAC3-miKO did downregulate PU.1 expression, which was proved to be responsible for gene regulation induced by HDAC3-miKO 30, the elevation of CCL7 expression in this study suggested another regulatory mechanism independent of PU.1 in HDAC3-miKO after stroke, which needs further investigation.

Another important question is what these macrophage reinforcements would function in the infarct core, which is always considered irrevocable and thus not a typical target after stroke. Since HDAC3-miKO promoted oligodendrogenesis and improved white matter repair, we hypothesized that these extra macrophages may be involved in stroke-induced myelin debris removal considering that phagocytosis of tissue debris is one of the core functions of macrophages. We first demonstrated the spatiotemporal distribution of myelin debris after tFCI, which has never been reported so far in mice. Our results showed that myelin debris was confined to the infarct core and peaked 3 to 7 days then completely disappeared as late as 14 days after tFCI. The spatial distribution of myelin debris coincided with infiltrated macrophages' residency, suggesting the possibility of myelin debris removal by macrophages. Indeed, a greater number of macrophages directly internalized a greater amount of myelin debris whereby their removal was finally accelerated in miKO-tFCI despite the unchanged phagocytosis capacity of macrophages. Of note, consistent with existing studies 55, infiltrated macrophages showed greater uptake or degradation capacity than microglia, suggesting macrophages as the predominant phagocyte in terms of myelin debris. Therefore, this accelerated removal of myelin debris by macrophage reinforcements may play an important role in improved remyelination in HDAC3-miKO following stroke, considering the fact that myelin debris contains a variety of inflammatory and neurotoxic factors that inhibit remyelination process 61. However, in addition to efficient myelin debris removal, remyelination also depends on cytokines for OPCs recruitment, proliferation and differentiation released by microglia 39. Follow-up studies should investigate whether the secretory products of HDAC3-deficient microglia affect oligodendrogenesis. Furthermore, tracking microglial changes at later timepoint (e.g. day 35 when we have observed ameliorated white matter injury in miKO-tFCI) could reveal other potential effects of HDAC-deficient microglia on white matter repair.

Additionally, another limitation of the current study is that only male mice were used in all experiments. This was done because male mice typically provide more consistent results in stroke models. However, since sex differences in ischemic stroke are well-documented 62, our exclusion of females means we did not examine the potential role of microglial HDAC3 in females, nor did we investigate possible sex-specific effects on stroke recovery and immune responses. Future studies should include both male and female mice to better understand the role of HDAC3 in stroke and how sex might influence the outcomes.

Of note, due to the channel limitations of conventional immunofluorescence in this study, despite the fact that there were far more peripherally infiltrating macrophages than microglia in the core, we still do not know yet whether the leftover microglia in the core are involved in myelin debris clearance and the extent of their involvement. Besides, while P2RY12 is a commonly used marker for homeostatic microglia, it is known to be downregulated in highly activated microglia, which may have led to an underrepresentation of activated microglia, especially in the infarct core. Given these limitations, future studies using additional specific macrophage/microglia markers, microglia-specific reporter mice, parabiosis surgery etc. are needed to more precisely identify the major cells that undergo myelin debris phagocytosis during ischemic stroke. Lampron *et al.* showed that limiting macrophage infiltration using CCR2^-/-^ transgenic mice did not affect cuprizone-induced demyelination 63, implying that microglia are sufficient to clear myelin debris in this model. However, in spinal cord injury, while microglia were the predominant cells in contact with axons up to 3 days after injury, macrophages then became the dominant cells in contact with degenerating axons instead as they infiltrated 64. They had more contents compared to microglia, and retained the contents until 42 days after injury with a persistent phagocytic function 64. Taken together, we conjecture that which cell type can be the mainstay of myelin debris phagocytosis is likely to depend on whether there is focal microglia loss during demyelination.

On the other hand, it is worth noting that although in this study, we have highlighted the role of infiltrated macrophages to myelin debris, there is still a lack of studies on the changes in macrophages themselves that have engulfed myelin debris, which also have complex and controversial effects on their scavengers 65, 66. Studies in spinal cord injury have shown that following degradation of myelin debris, macrophages turn into a foamy lipid-accumulating phenotype and secret neurotoxic lipids via FAK/PI3K/Akt/NF-κB signal pathway 67-69, shifting from an anti-inflammatory M2-like phenotype to a pro-inflammatory M1-like phenotype 68. In addition, these myelin-accumulating macrophages also suppressed OPCs' survival and thereby the remyelination process 67-69. Furthermore, macrophages or microglia laden with myelin debris will also have their response to subsequent stimuli reduced, inhibiting their further phagocytosis of myelin debris 70. However, in contrast to these findings, some studies revealed that in the core zone of multiple sclerosis accumulated a population of myelin-laden macrophages that expressed both M1-like and M2-like markers 71, 72. Liu *et al.* demonstrated that phagocytosis of myelin modulates activation of microglia pre-stimulated by interferon-γ (IFN-γ) or a combination of IFN-γ and lipopolysaccharide with a biphasic temporal pattern *in vitro*, i.e. the production of pro-inflammatory factors was promoted in the first phase (<6 h) but suppressed in the second phase (6-24 h) 73. These studies imply that the phenotypic shift of myelin-laden microglia or macrophages is closely dependent on when and where they are in the context of ischemic stroke. It is also reported that macrophage produced neurotrophic factors after stimulation by myelin debris 74. In the field of ischemic stroke, the ultimate fate of microglia or macrophage which have undergone removal of myelin debris remains to be further explored.

In our study, as we investigated the phagocytosis capacity of infiltrated macrophages via *ex vivo* phagocytosis assay, another unexpected truth emerged that HDAC3-deficient microglia showed significantly reduced capacity for uptake or degradation of myelin debris. Correspondingly, in the peri-infarct striatum where microglia accumulated and myelin sheath remained intact, we also observed less contact between Iba1^+^ cells and intact myelin bundles. A previous study has demonstrated that as stroke pathology progressed (until 7 days after a rat tFCI model), activated microglia/macrophages surrounded peri-infarct undamaged myelinated bundles, but did not phagocytic, unlike the Iba1^+^ cells in the infarct core that infiltrated into damaged bundles 75. However, Zhang *et al.* revealed that these microglia surrounding the myelin bundles showed CD68 (a lysosome marker) and CD86 (a pro-inflammatory marker) immunoreactivity and therefore they were considered active in phagocytosis of myelin that further aggravated white matter injury in chronic cerebral hypoperfusion 47. Recent studies also reported microglial trogocytosis (partial phagocytosis) of axon shafts in the developing mouse brain 76 and the *Xenopus laevis* retinotectal circuit 77. In this way, we inferred that the reduction of this contact induced by HDAC3-miKO may protect intact myelin fibers from secondary injury following stroke. However, it remains elusive whether microglia/macrophages are actually involved in phagocytosis, or trogocytosis of undamaged myelinated axon bundles after stroke and what signals drive activated microglia/macrophages to either phagocytose degraded myelin debris or just trogocytose intact myelin bundles. On the other hand, the mechanism by which HDAC3 deficiency inhibited microglial phagocytosis capacity is still unknown in our study. Interestingly, Greenhalgh *et al.* showed that microglial phagocytosis potential was negatively correlated with the number of infiltrating macrophages, and that co-culture of microglia and macrophages resulted in weaker phagocytosis of pHrodo-labelled myelin by microglia but stronger myelin phagocytosis by macrophages compared to culture of either microglia or macrophages alone 78. Indeed, in terms of spinal cord injury, inhibition of macrophage infiltration also promoted microglial phagocytosis 64. These studies partially support our findings. Nevertheless, future studies are needed to investigate the underlying molecular mechanism associated with HDAC3 in terms of microglial phagocytosis.

## 4. Conclusion

Overall, our study reveals that in the absence of HDAC3, microglia ultimately promote remyelination by recruiting macrophages and accelerating the removal of myelin debris with the benefit of these extra macrophages, highlighting the essential role of infiltrated macrophages in HDAC3-miKO-induced beneficial outcomes. This study provides a novel perspective as well as promising targets for white matter repair after ischemic stroke.

## 5. Experimental Section/Methods

*Experimental animals*: HDAC3-miKO animals were constructed as our previous study described 30. HDAC3^loxp/loxp^ mice were constructed by Shanghai Organism Model Center, while CX3CR1^CreER^ mice (RRID: IMSR_JAX: 021160) were provided by the Jackson Laboratory. By breeding HDAC3^loxp/loxp^ with CX3CR1^CreER^ mice, two mouse genotypes were finally generated: CX3CR1^wt/wt^: HDAC3^loxp/loxp^ (wild-type, WT) and CX3CR1^CreER/wt^:HDAC3^loxp/loxp^ (conditional knockout, HDAC3-miKO).

Only male mice were used throughout this study. Microglia-specific knockout of HDAC3 was induced by intraperitoneal injection of 100 mg/kg tamoxifen (T2859, Sigma-Aldrich) which is dissolved in a 9:1 mixture of corn oil and anhydrous ethanol for 5 consecutive days at the age of 6-8 weeks. To avoid off-target HDAC3 knockout on infiltrated macrophages, Sham or tFCI was performed at least one month later when circulating monocytes were nearly completely renewed by HDAC3-expressing progenitor cells.

All animals were housed in cages with individual ventilation systems, maintained at a temperature of 18-22°C and a humidity of 50-60%. The animals lived in a 12-hour light/12-hour dark cycle and had free access to food.

*Murine model of tFCI*: tFCI model was used to mimic ischemic stroke by occluding the left middle cerebral artery (MCA) for 60 min as previously described 79. In brief, mice were initially anesthetized with 5% isoflurane, then maintained under anesthesia with 1.5% isoflurane in a 30% O_2_ & 70% N_2_ mixture. An 8-0 monofilament with a silicon-coated tip was inserted into the left common carotid artery and advanced to the left MCA to restrict blood flow until a drop of more than 75% in blood flow as monitored by Laser Doppler Flowmetry (otherwise, the mouse was excluded). Rectal temperature was kept at 37.0 ± 0.5°C using a heating pad. The filament was removed after 60-min occlusion to achieve reperfusion. Sham-operated mice underwent the same procedure without MCA occlusion. Surgeries were conducted by a researcher blinded to the genotypes or experimental groups of the animals.

*Drug administration*: BrdU (5-Bromo-2'-deoxyuridine): Three days after tFCI, BrdU (19-160, Sigma-Aldrich) dissolved in phosphate-buffered saline (PBS) was administered intraperitoneally at a dosage of 50 mg/kg/day in the dark every 12 hours for 4 consecutive days.

### Neurofunctional evaluation

*Garcia score*: We employed an 18-point scoring system adapted from the one developed by Garcia et.al for stroke assessment as described by our previous study 30, 80. Garcia score was measured prior to the surgery and then repeatedly measured on designated days after tFCI.

*Foot fault test*: Mice underwent pre-training for 3 days prior to tFCI, freely walking for 5 minutes daily on a wire frame composed of 1.5 cm × 1.5 cm square grids. The grid measured 40 cm in length, 20 cm in width, and 30 cm in height. Measurements taken on the day before tFCI/Sham served as preoperative data. The test was repeatedly conducted on the designated days post-tFCI, with each test recorded for 5 minutes. The videos were then used to analyze foot fault rate by a researcher blinded to the experimental group. In details, the number of foot faults and total steps of the right forelimb (contralateral to the ischemic hemisphere) were counted from the second minute, and the foot fault rate was calculated as the number of foot faults divided by the total steps until the total steps reached 100. The same method was applied to the right hindlimb. This assessed sensorimotor dysfunction following tFCI in mice.

*Immunofluorescence and image analysis*: Under deep anesthesia, mice were perfused with pre-cooled 1×PBS then 4% paraformaldehyde solution (PFA, BL539A, Biosharp) for fixation. Brains were then removed and post-fixed in 4% PFA for 24 hours, followed by sequential gradient dehydration with 20% and 30% sucrose PBS solutions at 4°C. Subsequently, 25-μm-thick frozen serial coronal brain slices were prepared using a freezing microtome (Thermo Fisher) and then stored at -20°C in anti-freeze storage solution (30% ethylene glycol, 30% sucrose in phosphate-buffered solution).

These brain slices were used for immunofluorescence staining. Brain slices were rinsed in PBS and 0.3% Triton X-100 in PBS (PBST), followed by permeabilization using 1% PBST for 20 min. After wash for 3 times in 0.3% PBST, brain slices were blocked with QuickBlock™ blocking buffer (P0260, Beyotime) and then incubated with primary antibodies diluted in QuickBlock™ primary antibody dilution solution (P0262, Beyotime) overnight at 4°C. After that, slices were washed and incubated with secondary antibodies diluted in QuickBlock™ secondary antibody dilution solution (P0108, Beyotime) for 1 hour at room temperature. After washing, brain slices were mounted with DAPI Fluoromount-G® (0100-20, SouthernBiotech) and stored at -20°C. Of note, for P2Y12 staining, antigen retrieval was conducted prior to the blocking step. For BrdU staining, an additional DNA denaturation step (1 hour in 1N HCl at 37°C, followed by 10 min in 0.1M boric acid at room temperature, pH=8.5) was conducted prior to the blocking step.

Primary antibodies used in this study included: rabbit anti-P2Y12 (1:500, 69766S, CST), rat anti-F4/80 (1:500, 123101, Biolegend), goat anti-Iba1 (1:1000, ab5076, Abcam), rabbit anti-dMBP (1:1000, AB5864, Sigma-Aldrich), rabbit anti-HDAC3 (1:300, ab32369, Abcam), rat anti-BrdU (1:1000, ab6326, Abcam), mouse anti-Caspr (1:300, MABN69, Millipore), rabbit anti-Nav1.6 (1:300, ASC-009, Alomone), rabbit anti-NeuN-488 (1:1000, ab190195, Abcam), mouse anti-APC (1:300, OP-80, Millipore), rat anti-MBP (1:1000, ab7349, Abcam), rabbit anti-Iba1 (1:1000, 019-19741, Wako).

Secondary antibodies included: anti-rabbit secondary antibody conjugated with Alexa Flour 488 (1:1000, 711-545-152, Jackson ImmunoResearch Laboratories), anti-rabbit secondary antibody conjugated with Cy5 (1:1000, 711-605-152, Jackson ImmunoResearch Laboratories), anti-goat secondary antibody conjugated with Alexa Flour 488 (1:1000, 705-545-147, Jackson ImmunoResearch Laboratories), anti-mouse secondary antibody conjugated with Cy3 (1:1000, 115-165-146, Jackson ImmunoResearch Laboratories), anti-rat secondary antibody conjugated with Cy3 (1:1000, 712-165-153, Jackson ImmunoResearch Laboratories), anti-rat secondary antibody conjugated with Alexa Flour 488 (1:1000, 112-545-003, Jackson ImmunoResearch Laboratories).

All fluorescence images were captured using confocal microscopy (FV3000, Olympus). Specifically, Caspr/Nav1.6 images were captured with 60× oil objective. Other images for representation or analysis were all captured with 40× objective. For representative large images, images were captured with 10× objective and then stitched using Grid/collection stitching function in ImageJ. For z-stack confocal images, images were acquired using a fixed z-step of 0.5 μm with 40× objective.

Z-stack confocal images were imported into Imaris 10.0.1 (Bitplane) for 3D reconstruction. The generated 3D structures were used for measurement of dMBP^+^ myelin debris volume as well as the corresponding engulfment assay. These methods were adapted from the ones developed by Schafer et.al and Cignarella et.al 81, 82. First, 3D surface rendering was performed on the dMBP channel (Smoothing=0.3) using appropriate threshold. The total dMBP volume per unit was accordingly calculated as dMBP volume/the total volume of the field of view (FOV). For engulfment assay, 3D rendering was performed on the phagocytes channel (i.e. F4/80 channel) in the same way as mentioned above (Smoothing=0.6). DAPI^+^ phagocytes were maintained. Incomplete phagocytes that were located at the edges were excluded. Overlap volume ratio to the dMBP surface was then calculated by the software, demonstrating the ratio of engulfed dMBP volume to each phagocyte volume. “Engulfed” cells were defined as cells with overlap volume ratio greater than zero, while “no contact” cells had a ratio equal to zero. The average overlap volume ratio for each FOV is calculated as the mean of the overlap volume ratios of all cells within that FOV. 1-4 FOVs were analyzed for each mouse and the average overlap volume ratio of each mouse were computed. The volumes of phagocytes were also recorded and averaged as above. Overlap volume of each cell was recorded and summed to obtain the total engulfed dMBP volume within each FOV. The average of the values across all FOVs was then calculated to determine the total engulfed dMBP volume for each mouse.

All other image analyses were conducted using ImageJ.

*Flow cytometry*: Immune cell populations in the brain, peripheral blood and spleen were analyzed using flow cytometry 3 days after tFCI or Sham as previous studies described 40, 83. In brief, under deep anesthesia, mice were thoracotomized and injected with 1 ml of heparin sodium solution to prevent blood coagulation. Blood was then drawn and mixed with the anticoagulant. Subsequently, mice were perfused with pre-cooled 1× Hank's balanced salt solution (HBSS). Spleens and brains were harvested immediately afterwards.

*Brain tissue processing*: Brain tissue was minced and enzymatically digested using Neural Tissue Dissociation Kit (P) (130-092-628, Miltenyi Biotec) to produce a single cell suspension, which was then filtered through a 70 μm cell strainer. Single-cell suspensions were diluted to produce 30% Percoll suspensions, which were then slowly overlaid on 70% Percoll. After centrifugation (800 g, 30 min, 18°C, no brake and acceleration speed), the enriched brain-infiltrating leukocytes were collected from the 30%-70% interphase of Percoll gradient. After being washed with HBSS containing 2% fetal bovine serum (Thermo Fisher Scientific), the cell suspensions in the final 100 μl HBSS + 2%FBS were blocked on ice with rat anti-CD16/32 antibody (553142, BD Biosciences) for 10 min and then stained with fluorophore-conjugated antibodies in the dark at 4°C for 30 min. These antibodies included: CD45-efluor450 (48-0451-82, Thermo Fisher Scientific), CD11b-APC-Cy7 (47-0112-82, Thermo Fisher Scientific), CD3-APC (17-0032-82, Thermo Fisher Scientific), Gr1(Ly6G)-PE (12-9668-82, Thermo Fisher Scientific), Ly6c-BV605 (563011, BD Biosciences), CD11c-PerCP-Cy5.5 (45-0114-82, Thermo Fisher Scientific), CD19-FITC (11-0193-82, Thermo Fisher Scientific).

*Processing of peripheral blood and spleen samples*: Spleens were ground and filtered through a 70 μm cell strainer. Red blood cells in spleens or blood were lysed with ACK buffer (A1049201, Thermo Fisher Scientific). The remaining cell pellets after centrifugation (500 g, 5 min, 18°C) were processed similarly to the brain samples for blocking and staining.

Flow cytometry was performed on the Beckman Coulter CytoFLEX flow cytometer, with data analyzed using the FlowJo v10 software (BD Biosciences). Additionally, Cytek Aurora (Cytek® Biosciences) was used for cell sorting. Single staining of each antibody was prepared for compensation. The flow-sorted microglia (CD11b^+^CD45^int^) or macrophages (CD11b^+^CD45^hi^Ly6C^-^CD11c^-^) immediately underwent RNA extraction for qPCR or bulk RNA-seq.

*Ex vivo myelin phagocytosis assay*: Myelin was isolated from mice before *ex vivo* myelin phagocytosis assay according to a published protocol 84. Myelin was highly enriched by sucrose density gradient ultracentrifugation using the Optima XPN ultracentrifuge (Beckman Coulter). According to this protocol, we finally harvest ~143.3 mg myelin (wet weight) (~600 μg myelin protein) from 6 adult female mice. The purified myelin was dissolved in 180 μl cold 1×TBS containing protease inhibitor and stored at -80°C.

The conjugation of myelin with pHrodo-Red (P36600, Thermo Fisher Scientific) was produced referring to the manufacturer's instruction and a published protocol 85. 92.5 μl myelin was first mixed with 25 μl pHrodo-Red in a 1.5 ml centrifuge tube. The mixture was then resuspended in 132.5 μl PBS (pH 8). After 45 min incubation at room temperature in the dark, the suspension was centrifuged for 10 min at 4000 g, 4°C. Discard the supernatant and resuspend the remaining pellet in 475 μl 1×PBS (pH 7.2). The pHrodo-labelled myelin was stored at -80°C.

Myelin was also labelled PKH26 (PKH26GL, Sigma-Aldrich) following the manufacturer's instruction. 30 μl myelin dissolved in TBS was centrifuged for 5 min at 400 g into a loose pellet. The suspension was discarded and remaining fluid was removed, ensuring that no more than 25 μl was left. Subsequently, 1 ml Diluent C was added to the pellet to prepare a 2×myelin suspension followed by gentle pipetting. Immediately prior to staining, prepare a 2×Dye Solution in Diluent C by adding 4 μl of the PKH26 ethanolic dye solution to 1 ml of Diluent C and mix well. The 1 ml of 2×myelin suspension was rapidly added to the 1 ml of 2×Dye Solution. After being mixed by pipetting, the myelin/dye suspension was incubated for 5 min with periodic mixing. The staining was stopped by adding an equal volume (2 ml) of 1% BSA. Incubate for 1 min to allow binding of excess dye. Finally, wash the myelin pellet 3 times with 10 ml 1×HBSS each time by centrifugation at 400 g, 20-25°C for 10 min. After the final wash, the myelin pellet was resuspended in 200 μl 1×HBSS and stored at -80°C.

Our method for evaluating myelin phagocytosis by microglia/macrophages was adapted with minor modifications from the one described by Gómez-López et.al 85. To enrich brain-infiltrating leukocytes, we employed the same Percoll gradient centrifugation protocol as described in our method section of flow cytometry. The collected leukocytes from the interphase layer were added to 10 ml HBSS+10% FBS, followed by centrifugation for 10 min at 800 g, 24°C with maximum brake speed. Discard the supernatant and resuspend pellet in 100 μl DMEM F-12 (11320-033, Thermo Fisher Scientific) + 10% FBS. PKH26/pHrodo-Red-labelled myelin was thawed and vortexed for 1 min. After that, 3 μl of labelled myelin was added to the 100 μl cell suspension in DMEM F-12. The tube without labelled myelin served as negative controls for phagocytosis. Incubate the samples for 4 hours at 37°C in a CO_2_ cell culture incubator (HF90, Heal Force) without agitation in the dark. After incubation, the suspension was filled with PBS to 15 ml, followed by centrifugation for 5 min at 310 g, 24°C. The blocking and the subsequent staining steps were performed as described before (the flow cytometry section). We used CD45-eFluor450 (48-0451-82, Thermo Fisher Scientific) and CD11b-APC-Cy7 (47-0112-82, Thermo Fisher Scientific) antibodies to distinguish microglia and macrophages. The flow cytometry data acquisition was conducted in the Beckman Coulter CytoFLEX flow cytometer. PE channel (488 nm excitation, 585/42 BP channel) was used for detection of PKH26/pHrodo-Red signals. The myelin-laden cell population was gated according to the negative control.

### Macrophage reconstitution with CCR2-deficient BMDMs in mice

*Bone marrow-derived macrophage (BMDM) culture:* CCR2-KO mice (Catalog No. NM-KO-190018) or c57/BL6 (WT, CCR2^+/+^) mice were obtained from Shanghai Model Organisms Center, Inc. Bone marrow-derived cells were isolated and differentiated into macrophages as previously described 48. Briefly, CCR2-KO mice or WT mice (6-8 weeks old) were euthanized by cervical dislocation and immediately immersed in 75% ethanol for sterilization. The femur and tibia were carefully isolated, and any muscle tissue was removed. Both ends of the long bones were cut, and the bone marrow cavity was flushed with sterile D-PBS using a syringe to collect the bone marrow cells. Red blood cells were lysed using ACK lysis buffer (A10492-01, Gibco). The remaining cells were resuspended in RPMI-1640 medium (11875093, Gibco) supplemented with 10% FBS (10099141C, Gibco), 1% Penicillin-Streptomycin (15140-122, Gibco), and 5 ng/mL M-CSF (HY-P7085, MCE). A total of 10^7^ cells were seeded into 10-cm culture dishes and incubated at 37°C with 5% CO2. The medium was replaced every 2-3 days. After 5 days of culture, macrophage differentiation was confirmed by flow cytometry.

*Macrophage depletion and reconstitution:* To deplete peripheral macrophages, 0.2 mL of clodronate liposomes (Liposoma, C-005) was injected into the tail vein of the mice every 24 hours for 2 days prior to tMCAO. After tMCAO, macrophage-depleted mice were reconstituted with cultured BMDMs via tail vein injection 2 hours post-stroke. Three experimental groups were used in this section: 1) WT mice reconstituted with WT BMDMs (WT BMDM + WT-tFCI), 2) HDAC3-miKO mice reconstituted with WT BMDMs (WT BMDM + miKO-tFCI), and 3) HDAC3-miKO mice reconstituted with CCR2^-/-^ BMDMs (CCR2^-/-^ BMDM + miKO-tFCI).

*Quantitative real-time PCR*: Total RNA of the flow-isolated microglia or macrophages was extracted using RNeasy Plus Micro Kit (74034, QIAGEN) following the manufacturer's instruction. The first-strand cDNA was generated using reverse transcriptase-PCR with the PrimeScript RT Reagent Kit with cDNA Eraser (Perfect Real Time) (RR047A, Takara Bio) according to the manufacturer's instruction. cDNA was diluted to an appropriate concentration using RNase-free water and stored at -20°C until use. The real-time PCR reaction solution in a 384-well contains the following components: 5 μl Hieff QPCR SYBR Green Master Mix (11201ES08, Yeasen), 2 μl cDNA, 0.2 μl forward primer, 0.2 μl reverse primer. The reaction was performed at 95°C for 5 min, followed by 40 cycles of 95°C for 10 s, 55°C for 20 s, and 72°C for 20 s on a QuantStudio 5 Real-Time PCR system (Thermo Fisher Scientific). The primer sequences used in this study are shown in Table 1.

*RNA sequencing and analysis*: The library preparation of flow-sorted microglia and the paired-end RNA sequencing was performed by Shanghai Meigiddo Biological Pharmaceutical Co.Ltd as described in our previous study 30. Each group included 3 biological replicates, each of which was a combination of microglia from 3-4 ischemic hemisphere. The pre-processing steps of the RNA-seq data, including quality check, adaptor trimming, alignment to the genome, and count generation, and the differential expression genes analysis with batch effects removed in R have been described in the previous study 30. Importantly, differential gene expression was conducted using the R package DESeq2. To remove batch effect, we then employed the removeBatchEffect function from the limma package to adjust the normalized counts. In this study, we performed pairwise comparison between miKO-tFCI to WT-tFCI. Genes with adjusted p value < 0.05 and |log2FC| > 0.58 were regarded as DEGs. Shrunk fold change produced by an adaptive shrinkage estimator ashr was used for visualizing and ranking of genes in the following analysis 86.

Then we used the gseGO function in the clusterProfiler package 87 to perform GSEA 88 on the aforementioned DEGs based on Gene Ontology (GO) database. The ridgeplot function was employed for visualize the results of GSEA. The expression of genes annotated to the term “Leukocyte Chemotaxis”, which was derived from the GSEA result in all replicates, was visualized as heat map using the ComplexHeatmap package 89, with leukocyte chemokines indicated on the graph.

The bulk RNA-seq data are available and can be accessed with GEO accession GSE220042.

*DTI*: DTI is particularly useful for assessing the white matter integrity. An 11.7 animal magnetic resonance imaging (MRI) system (Bruker BioSpec 117/16) with a 6 mm 4-channel surface array coil (Bruker BioSpin) was used for DTI. DTI was performed* in vivo* on day 3 and 14 post tFCI using an Echo Planar Imaging (EPI)-DTI sequence with the following parameters: TR/TE = 2500/30 ms, Direction = 30, FOV = 20 × 20 mm, acquisition matrix = 200 × 200, 30 slices with a slice thickness of 0.5 mm, 3 averages, 4 segments, b-value = 3,000 s/mm^2^.

The* ex vivo* scan was conducted 35 days post-tFCI using the following procedure. Mice were perfused with PBS followed by PFA, after which the brains were extracted through decapitation and post-fixed in PFA for 24 hours. Following a PBS rinse to remove PFA, the brains were air-dried and put into 5-ml test tubes filled with carbon-free oil before being placed in the coil for scanning. The scanning parameters for the *ex vivo* DTI included: SE sequence 60 directions, TR/TE = 2800/17.5 ms, FOV = 16 × 16 mm, acquisition matrix = 80 × 80, 74 slices with a slice thickness of 0.2 mm, 3 averages, 4 segments, b-value = 650 s/mm^2^.

DTI data were analyzed using DSI Studio software (http://dsi-studio.labsolver.org/). Regions of interest (ROIs), including the EC and IC of both the ipsilateral and contralateral hemispheres, were manually delineated by a researcher blinded to the experimental groups to determine the average FA and RD within these ROIs. Directionally Encoded Color maps (DEC maps), FA, and RD maps were generated by DSI Studio software.

*Measurement of compound action potentials*: On 35 days after tFCI or Sham, mice were deeply anesthetized and perfused with artificial cerebrospinal fluid (124 mM NaCl, 2.5 mM KCl, 2 mM CaCl_2_, 1 mM NaH_2_PO4, 24 mM NaHCO_3_, 1.3 mM MgSO_4_ and 10 mM D-glucose). The brains were harvest and incubated in the same fluid at 32°C for 0.5 hours and then at room temperature for 1 hour to recover. 350-μm-thick coronal slices at Bregma -1.46 mm were collected using a vibrating microtome (VT1220S, Leica). A bipolar stimulation electrode was used to deliver a series of stimulation currents starting from 0 mA and increasing in 0.25 mA increments up to 2 mA at ~1 mm from the midline of the corpus callosum (CC). Glass microelectrodes, pre-filled with artificial cerebrospinal fluid and with resistances of 5-8 MΩ, recorded compound action potentials (CAPs) at 1 mm from the stimulation site in the EC. The amplitudes of the N1 (representing the activity of rapidly conducting myelinated fibers) and N2 (representing the activity of slow-conducting unmyelinated fibers) components of the CAPs were quantified using pClamp 10 software (Molecular Devices).

*Transmission electron microscopy*: Ultrastructural observations of white matter remyelination in the CC/EC area were conducted using transmission electron microscopy (TEM), based on the methods described in published studies 40, 90, 91. After CAPs measurement, the coronal brain sections were fixed for 24 hours in PBS containing 2.5% glutaraldehyde. The specific CC/EC regions near the infarct core were micro-dissected into 1 mm^3^ blocks. These samples were washed three times with 0.1 M PBS for 15 min each, and then fixed in 1% OsO_4_, 1% K_3_Fe(CN)_6_ for 2-3 hours. Subsequently, the samples were dehydrated in a series of ethanol and acetone washes—50%, 70%, 90% ethanol, a 1:1 mixture of 90% ethanol and 90% acetone, 90% acetone, and then three times of 100% acetone—each for 20 min at 4°C. For embedding, the samples were first pre-embedded in a 2:1 mixture of acetone and embedding resin for 4 hours at room temperature, followed by an overnight embedding in a 1:2 mixture, and finally embedded in the resin at 37°C overnight, then at 45°C for 12 hours, and finally at 60°C for 24 hours to ensure complete polymerization. Ultrathin sections (50-60 nm) were cut using a Leica LKB-1 ultramicrotome and stained with 3% uranyl acetate and lead citrate. Images were captured using a Philips CM120 transmission electron microscope and analyzed quantitatively with ImageJ software. 4-5 images (covering a total area of ~600 μm^2^) were collected per mouse to analyze the ratio of myelinated axons, and the g-ratio (ratio of the inner to outer diameter of fibers) were calculated for at least 250 randomly selected axons.

*Statistical analysis*: Statistical analysis was conducted using GraphPad Prism 10.1.2 software (Dotmatics), with all data presented as mean ± standard error of the mean (SEM). Normality of the data was tested using the Shapiro-Wilk test. Data conforming to normal distribution were analyzed with parametric tests; non-normally distributed data were analyzed using non-parametric tests. For comparisons between two groups, an unpaired two-tailed Student's t-test was used if both datasets were normally distributed; otherwise, the Mann-Whitney U test was employed. For data involving three or more groups, if all groups were normally distributed, an ordinary one-way analysis of variance (ANOVA; F-test) was used to assess mean differences, with Bonferroni post-tests (unless otherwise specified). If any group did not meet the normality assumption, the Kruskal-Wallis test was applied. For data with repeated measures over time or across stimulus intensities, the differences in mean values between groups were analyzed using two-way ANOVA with Bonferroni's multiple comparisons post-hoc test. Main effects in two-way ANOVA were calculated to compare overall differences between groups. Pearson's correlation analysis was utilized for correlation tests. Correlations between DTI data and behavioral data were computed using the cor function in R and visualized using heat maps drawn with the corrplot package. In all the aforementioned analysis, a *p*-value of <0.05 was considered statistically significant.

### Author Contributions

Yanqin Gao designed this study. Chenran Wang, Yue Zhang, Jiaying Li, Yichen Huang, Ziyu Shi, Yuwen Zhang, Leilei Mao, Yana Wang, Shuning Chen, and Yiwen Yuan performed experiments. He Wang critically checked the MRI. Jiaying Li and Yue Zhang analyzed the data. Jiaying Li and Leilei Mao wrote the manuscript. Yanqin Gao and Leilei Mao critically edited the manuscript. The authors read and approved the final manuscript.

## Supplementary Material

Supplementary figures.

## Figures and Tables

**Table 1:** qPCR primer sequences

Gene		Sequence (5'-3')
*Il1b*	forward	CTCCATGAGCTTTGTACAAGG
	reverse	TGCTGATGTACCAGTTGGGG
*Nos2*(iNOS)	forward	CAAGCACCTTGGAAGAGGAG
	reverse	AAGGCCAAACACAGCATACC
*Fcgr3a*(CD16)	forward	TTTGGACACCCAGATGTTTCAG
	reverse	GTCTTCCTTGAGCACCTGGATC
*Fcgr2a*(CD32)	forward	AATCCTGCCGTTCCTACTGATC
	reverse	GTGTCACCGTGTCTTCCTTGAG
*Cd86*	forward	GACCGTTGTGTGTGTTCTGG
	reverse	GATGAGCAGCATCACAAGGA
*Mrc1*(CD206)	forward	CCTTTCAGTCCTTTGCAAGC
	reverse	TGCGCTTGCAGAGATTAAAA
*Tgfb1*	forward	TGCGCTTGCAGAGATTAAAA
	reverse	CGTCAAAAGACAGCCACTCA
*Gapdh*	forward	CTGCCCAGAACATCATCCCT
	reverse	TGAAGTCGCAGGAGACAACC
*Chil3*(YM1/2)	forward	CAGGGTAATGAGTGGGTTGG
	reverse	CACGGCACCTCCTAAATTGT
*Arg1*	forward	TCACCTGAGCTTTGATGTCG
	reverse	CTGAAAGGAGCCCTGTCTTG
*Tnf*	forward	GACCCTCACACTCAGATCATCTTCT
	reverse	CCTCCACTTGGTGGTTTGCT
*Il13*	forward	CCTGGCTCTTGCTTGCCTT
	reverse	GGTCTTGTGTGATGTTGCTCA
*Ccl5*	forward	GGAGTATTTCTACACCAGCAGCAAG
	reverse	GGCTAGGACTAGAGCAAGCAATGAC
*Ccl2*	forward	CACTCACCTGCTGCTACTCA
	reverse	GCTTGGTGACAAAAACTACAGC
*Ccl3*	forward	CATGACACTCTGCAACCAAGTCTTC
	reverse	GAGCAAAGGCTGCTGGTTTCA
*Ccl7*	forward	GCTGCTTTCAGCATCCAAGTG
	reverse	CCAGGGACACCGACTACTG
*Ccl22*	forward	CTGATGCAGGTCCCTATGGT
	reverse	GCAGGATTTTGAGGTCCAGA
*Hdac3*	forward	CATCGCCTGGCATTGACTCAT
	reverse	AAGGCATTAAGGCTCTTGGTG
