# Supplementary material for: Orchestrating the frontline: HDAC3-miKO recruits macrophage reinforcements for accelerated myelin debris clearance after stroke
Source: Theranostics. 2025 Jan 1;15(2):632–55. doi: 10.7150/thno.103449 (PMC11671378; doi:10.7150/thno.103449)
Supplement: Supplementary file 1 — Supplementary figures. [file thnov15p0632s1.pdf]

Supplementary material

Orchestrating the frontline: HDAC3-miKO recruits macrophage reinforcements for accelerated myelin debris clearance after stroke

Jiaying Li†, Chenran Wang†, Yue Zhang†, Yichen Huang, Ziyu Shi, Yuwen Zhang, Yana Wang, Shuning Chen, Yiwen Yuan, He Wang, Leilei Mao, Yanqin Gao**

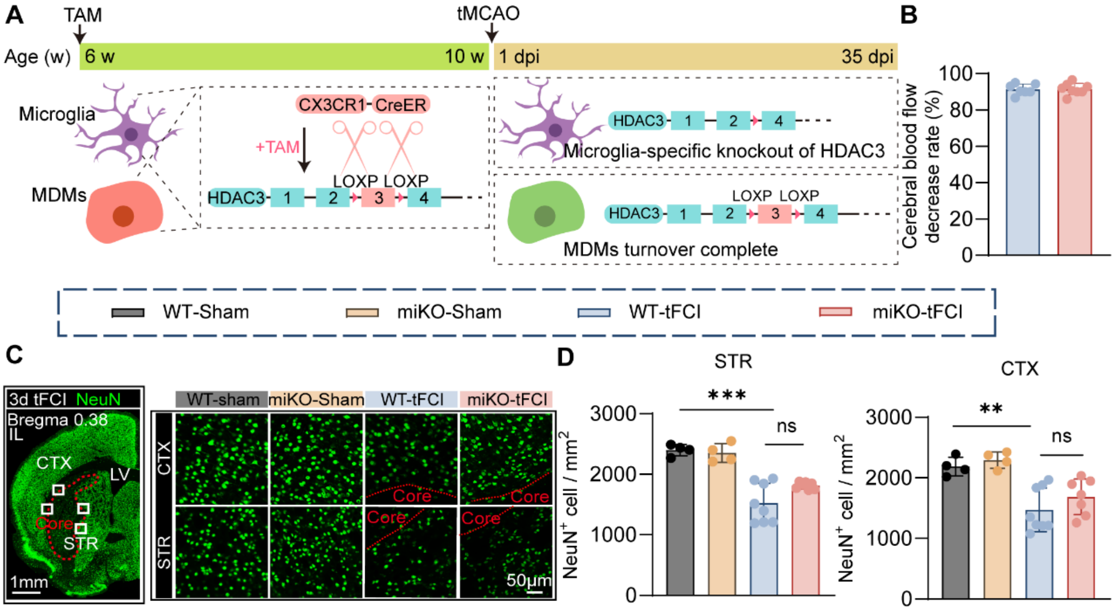

Figure S1. tFCI induced comparable decrease of regional cerebral blood flow in WT and HDAC3-miKO mice. (**A**) Schematic diagram showing the construction of microglia-specific HDAC3 knockout mouse line. (**B**) Decrease rates of cerebral blood flow in WT and HDAC3-miKO mice were comparable as detected by Laser Doppler flowmetry. n = 7 for WT-tFCI; n = 8 for miKO-tFCI. (**C**) Representative images of NeuN immunofluorescence staining 3 days after tFCI. White boxes (left panel) indicated where the images (right panel) were captured and analyzed. (**D**) Quantification of the number of NeuN$^+$ cells in the peri-infarct STR or CTX. n = 4 for WT-/miKO-Sham; n = 7 for WT-tFCI; n = 8 for miKO-tFCI. All data are presented as means±SEM. Data were analyzed using (B) unpaired two-tailed Student's t test or (D) one-way ANOVA followed by Bonferroni's post hoc test. ***p* < 0.01, ****p* < 0.001, ns: no significance, as indicated

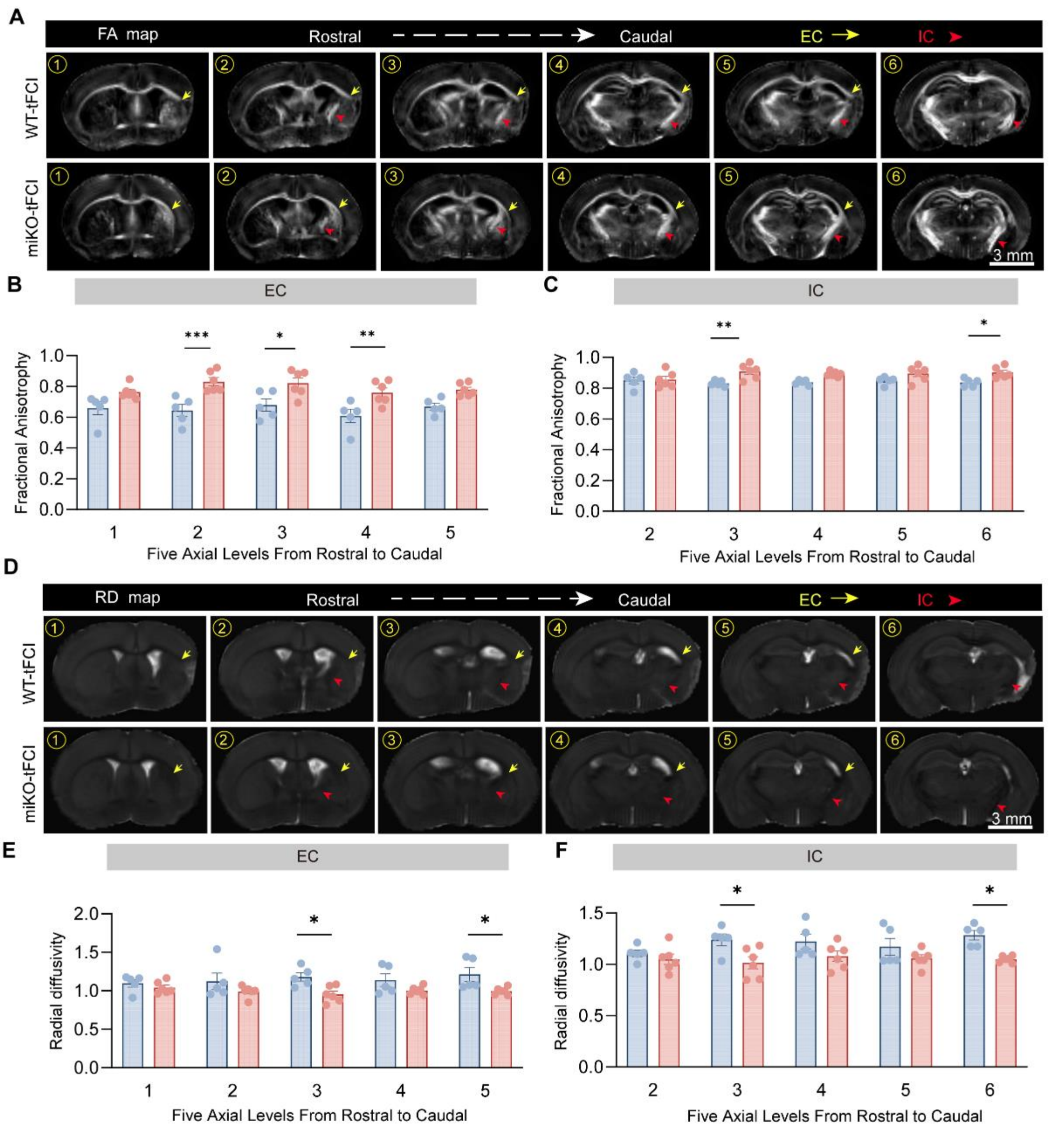

Figure S2. ***ex vivo*** **DTI-MRI on day 35 after tFCI, related to Figure 2.** (**A**) FA map of ex vivo DTI-MRI presented from rostral planes to caudal planes on day 35 after tFCI. (**B&C**) Quantification of FA value on different planes in the EC (B) or IC (C). (**D**) RD map of ex vivo DTI-MRI presented from rostral planes to caudal planes on day 35 after tFCI. (**E&F**) Quantification of RD value on different planes in the EC (E) or IC (F). All data are presented as means±SEM. All data were analyzed using two-way ANOVA followed by Bonferroni's post hoc test. *$p < 0.05$, **$p < 0.01$, ***$p < 0.001$, as indicated.

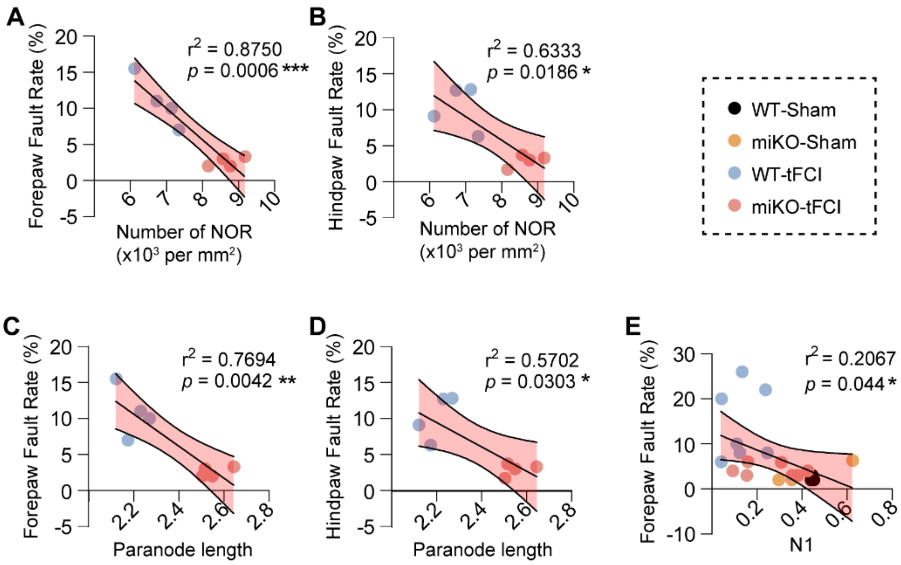

Figure S3. Correlation analysis, related to Fiugre 2. (**A-D**) Behavior test correlated to the number of NOR (A&B) or Paranode length (C&D). n = 4 for WT-/miKO-tFCI. (E) Behavior test correlated to N1 amplitude. n = 7 for WT-/miKO-tFCI; n = 3 for WT-/miKO-Sham. Data were analyzed using Pearson correlation. **p* < 0.05, ***p* < 0.01, ****p* < 0.001.

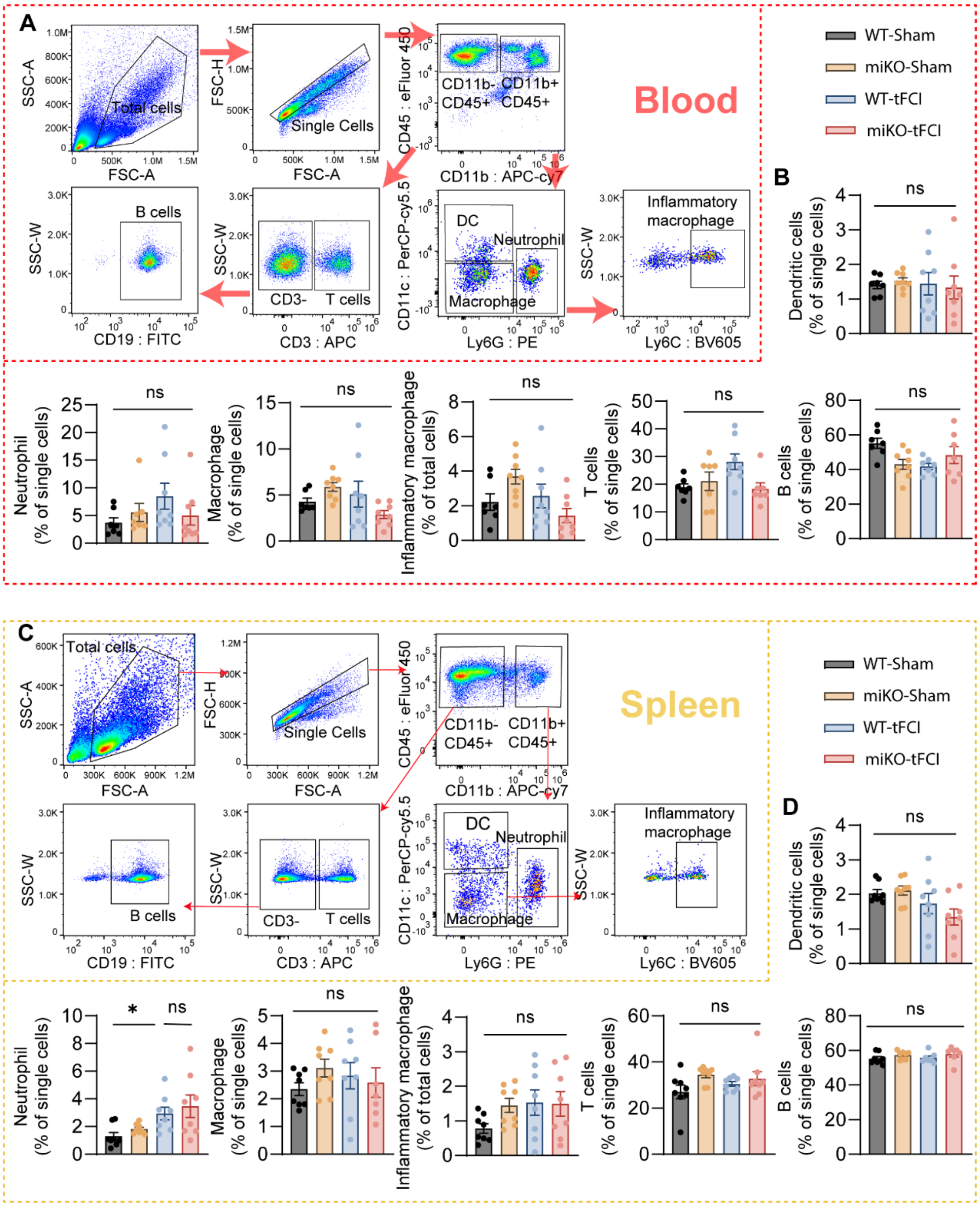

Figure S4. HDAC3-miKO did not affect immune response in peripheral blood and spleen 3 days after tFCI, related to Figure 4. (**A&B**) Gating strategy for blood sample (A) and the corresponding quantification (B, the percentage of the cell number to the number of single cells). (**C&D**) Gating strategy for blood sample (C) and the corresponding quantification (D, the percentage of the cell number to the number of single cells). All data are presented as means±SEM. Data were analyzed using one-way ANOVA followed by Bonferroni's post hoc test or Kruskal-Wallis test followed by Dunn's post hoc test. *$p < 0.001$, ns: no significance, as indicated.

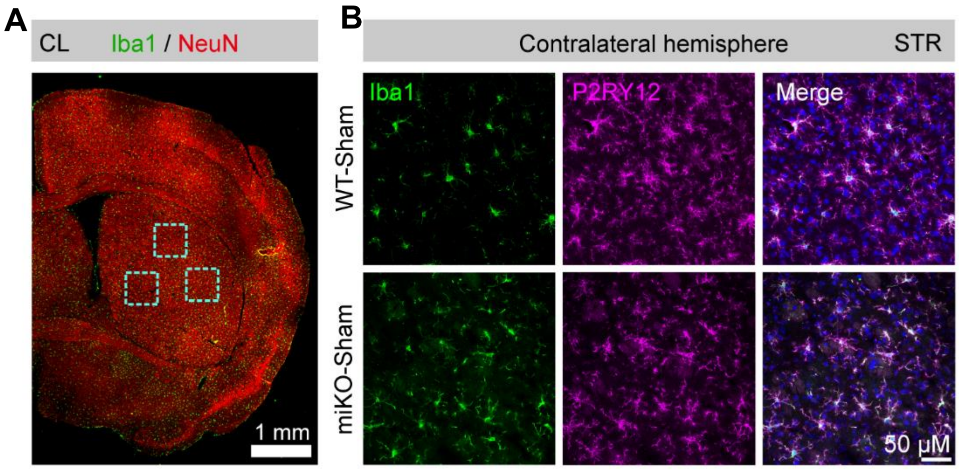

Figure S5. HDAC3-miKO did not change the number of microglia in the Sham group, related to Figure 4. (**A**) Representative large image of Iba1/NeuN in the contralateral hemisphere. Cyan boxes indicated where the (B) captured region in the STR. (**B**) Representative images of Iba1/P2RY12 immunofluorescence staining.

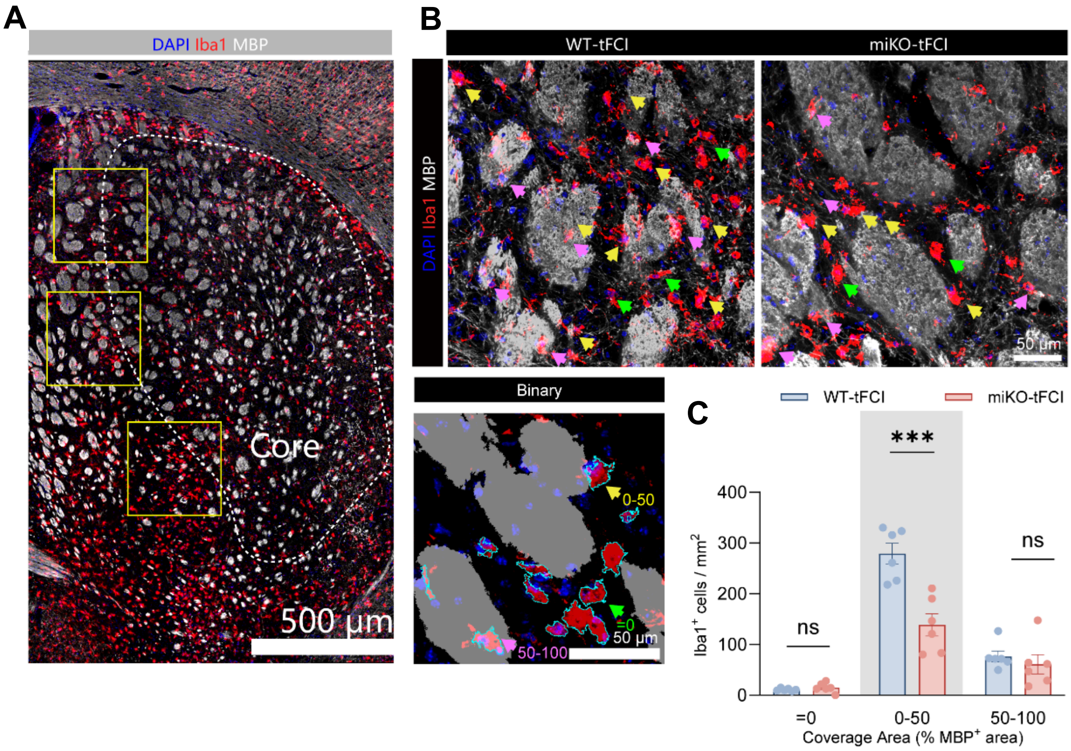

Figure S6. HDAC3-deficient microglia showed diminished contact with intact myelin sheath in the peri-infarct STR, related to Figure 6. (**A**) Representative large image of Iba1/MBP immunofluorescence staining with yellow boxes indicating the captured regions for quantification of contact between Iba1$^+$ cells and MBP$^+$ myelin sheath. (**B**) Upper panel: representative images of Iba1/MBP immunofluorescence staining. Lower panel: representative binary image. MBP channel was made binary and underwent appropriate median filter, followed by the removal of MBP$^+$ tiny filaments to allow the quantification of the contact between Iba1$^+$ cells (as outlined by cyan lines) and the intact myelin bundles. In this way, we measured the proportion of the Iba1$^+$ area

covered by the binary myelin bundle to the whole area of the Iba1$^+$ cell as coverage area. Green arrows indicated the Iba1$^+$ cells with coverage area equal to zero (i.e. no contact). Yellow arrows indicated the Iba1$^+$ cells with coverage area ranging from 0 to 50. Purple arrows indicated the Iba1$^+$ cells with coverage area ranging from 50 to 100. (**C**) Quantification of the number of Iba1$^+$ cells with different levels of coverage area. All data are presented as means±SEM. Data were analyzed using unpaired two-tailed Student's t test or Mann-Whitney test. ***$p < 0.001$, ns: no significance, as indicated.

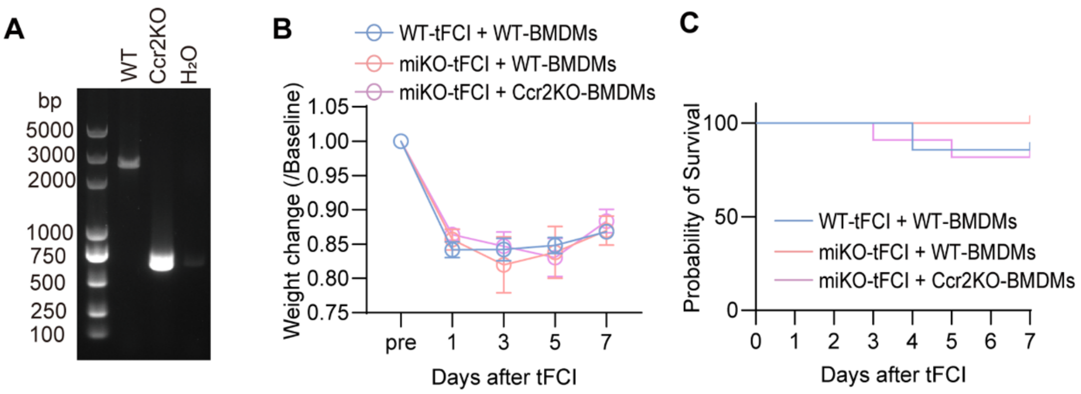

Figure S7. CCR2-KO BMDMs reconstitution did not change body weight and survival rate in HDAC3-miKO mice after tFCI, related to Figure 7. (**A**) PCR showing knockout of CCR2. **(B)** Body weight. **(C)** Survival rate. No significant change was observed.

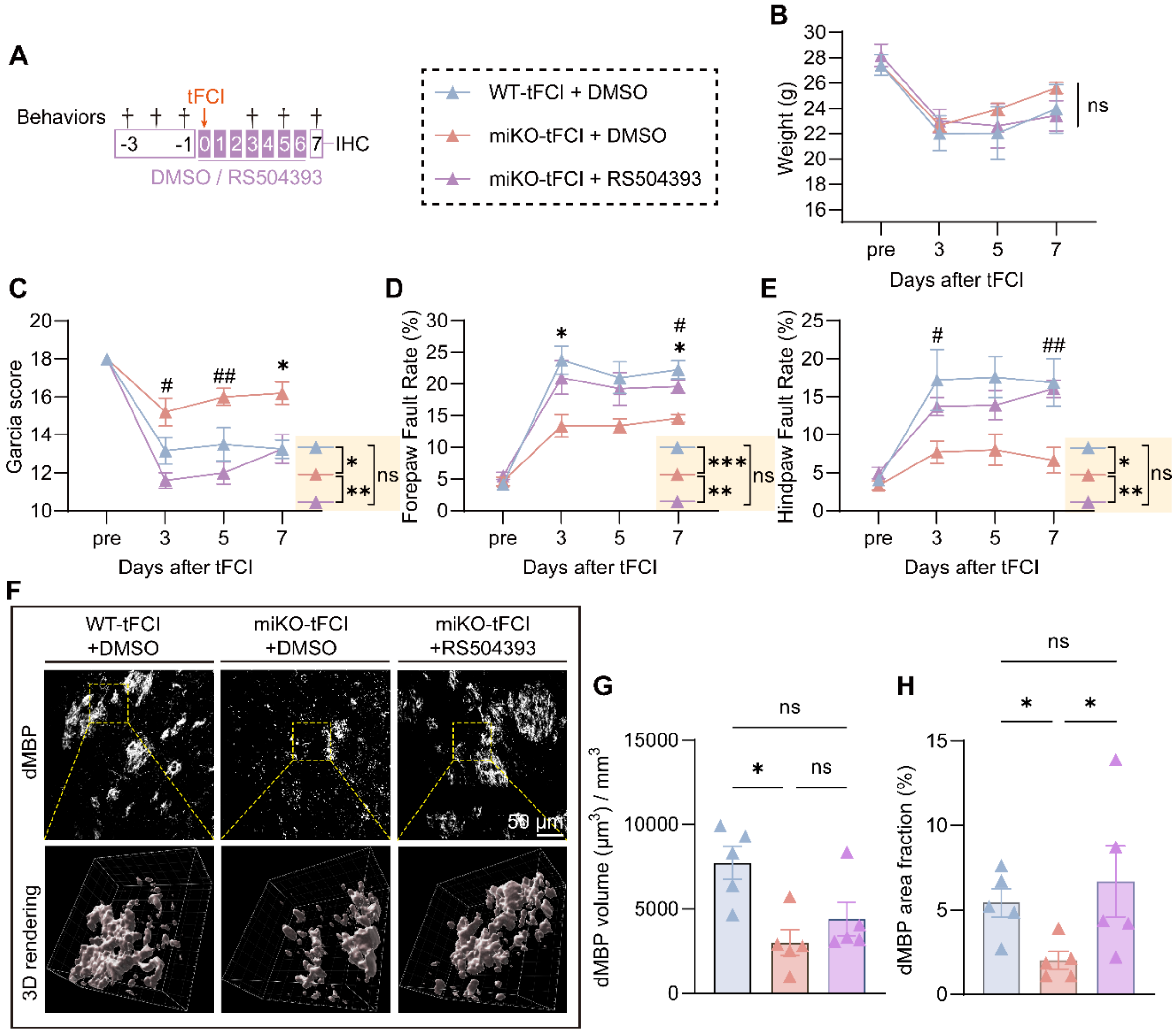

Figure S8. Blockade of CCR2 reverses the effects of HDAC3-miKO. (A) Experimental design indicating the strategy for DMSO or RS504393 administration. **(B)** Body weight. **(C-E)** Sensorimotor function assessment by Garcia score (C) and foot fault test (D&E). n = 6 for WT-tFCI+DMSO; n = 5 for miKO-tFCI+DMSO or miKO-tFCI+RS504393. **(F)** Representative images of dMBP immunofluorescence staining and the corresponding 3D rendering in three groups. **(G)** Quantification of dMBP volume (μm3) per mm3. **(H)** Quantification of dMBP area fraction (%). n = 5 for WT-tFCI+DMSO; n = 5 for miKO-tFCI+DMSO; n = 5 for miKO-tFCI+RS504393(G&H). All data are presented as means±SEM. Data were analyzed using (B-E) two-way ANOVA followed by Bonferroni's post hoc test. $^{*}p < 0.05$ for post hoc comparison between WT-tFCI+DMSO and miKO-tFCI+DMSO; $^{\#}p < 0.05$; $^{\#\#}p < 0.01$ for post hoc comparison between miKO-tFCI+DMSO and miKO-tFCI+RS504393. Comparisons in the yellow blocks referred to the main effects between groups for the ANOVA. (G&H) Kruskal-Wallis test followed by Dunn's post hoc test. $^{*}p < 0.05$, $^{**}p < 0.01$, $^{***}p < 0.001$, ns: no significance, as indicated.
